# Epigenetic factor siRNA screen during primary KSHV infection identifies novel host restriction factors for the lytic cycle of KSHV

**DOI:** 10.1371/journal.ppat.1008268

**Published:** 2020-01-10

**Authors:** Nenavath Gopal Naik, Thomas Hong Nguyen, Lauren Roberts, Luke Todd Fischer, Katherine Glickman, Gavin Golas, Bernadett Papp, Zsolt Toth

**Affiliations:** 1 Department of Oral Biology, University of Florida College of Dentistry, 1395 Center Drive, Gainesville, FL, United States of America; 2 UF Genetics Institute, Gainesville, FL, United States of America; 3 UF Health Cancer Center, Gainesville, FL, United States of America; 4 UF Informatics Institute, Gainesville, FL, United States of America; University of Southern California, UNITED STATES

## Abstract

Establishment of viral latency is not only essential for lifelong Kaposi’s sarcoma-associated herpesvirus (KSHV) infection, but it is also a prerequisite of viral tumorigenesis. The latent viral DNA has a complex chromatin structure, which is established in a stepwise manner regulated by host epigenetic factors during *de novo* infection. However, despite the importance of viral latency in KSHV pathogenesis, we still have limited information about the repertoire of epigenetic factors that are critical for the establishment and maintenance of KSHV latency. Therefore, the goal of this study was to identify host epigenetic factors that suppress lytic KSHV genes during primary viral infection, which would indicate their role in latency establishment. We performed an siRNA screen targeting 392 host epigenetic factors during primary infection and analyzed which ones affect the expression of the viral replication and transcription activator (RTA) and/or the latency-associated nuclear antigen (LANA), which are viral genes essential for lytic replication and latency, respectively. As a result, we identified the Nucleosome Remodeling and Deacetylase (NuRD) complex, Tip60 and Tip60-associated co-repressors, and the histone demethylase KDM2B as repressors of KSHV lytic genes during both *de novo* infection and the maintenance of viral latency. Furthermore, we showed that KDM2B rapidly binds to the incoming viral DNA as early as 8 hpi, and can limit the enrichment of activating histone marks on the RTA promoter favoring the downregulation of RTA expression even prior to the polycomb proteins-regulated heterochromatin establishment on the viral genome. Strikingly, KDM2B can also suppress viral gene expression and replication during lytic infection of primary gingival epithelial cells, revealing that KDM2B can act as a host restriction factor of the lytic cycle of KSHV during both latent and lytic infections in multiple different cell types.

## Author summary

Latent viral infection of cancer cells in KSHV-associated tumors is critical for the growth and survival of the cancer. Thus, revealing how lytic viral genes get suppressed through epigenetic regulation following *de novo* KSHV infection, resulting in the establishment of latency, is central to understanding the pathogenesis of KSHV infection. Importantly, the epigenetic factors that we identified as suppressors of KSHV lytic genes are not only crucial for the establishment and maintenance of KSHV latency in different cell types, but also several of them can block lytic KSHV infection in oral epithelial cells. Since herpesviruses often rely on similar sets of host epigenetic factors, the characterization of these newly identified epigenetic factors in KSHV infection may help to better understand fundamental epigenetic mechanisms that may also be utilized by other herpesviruses to establish latency following primary infection.

## Introduction

Kaposi’s sarcoma-associated herpesvirus (KSHV) is an oncogenic virus that causes lifelong infection in humans by alternating between latency and a lytic cycle in various cell types [1]. The establishment of viral latency following primary infection is particularly important in terms of KSHV pathogenesis. While latent virus can limit immune detection thereby promoting the persistent infection of the host, latently infected B cells and endothelial cells can also serve as progenitors of KSHV-induced cancers such as primary effusion lymphoma (e.g. BCBL1) and Kaposi’s sarcoma [2,3].

During latency, the KSHV DNA is maintained as a chromatinized episome in the nuclei of infected cells, which is regulated by host epigenetic factors. Some of the epigenetic factors support constitutive expression of latent genes, while others can block the expression of lytic genes [4,5]. The chromatin of latent genes were demonstrated to be enriched in activating histone marks (H3K4me3 and hyperacetylated histones), favoring gene transcription such as in the case of the latency-associated nuclear antigen (LANA), whereas the majority of lytic genes are heterochromatinized and enriched in repressive histone marks (e.g. H3K27me3, H3K9me3, and hypoacetylated histones) [6,7]. The repression of lytic genes during latency can be achieved by various inhibitory epigenetic and transcriptional mechanisms such as DNA methylation [8], Polycomb Repressive Complex 1 (PRC1)- and PRC2-mediated histone modifications [6,7,9], histone deacetylation [10,11,12,13], viral chromosome looping [14,15], and NELF-mediated transcription elongation inhibition [16]. Of the lytic genes, the transcriptional regulation of ORF50, which encodes the viral replication and transcription activator (RTA), has been studied in more detail because of its essential role in the lytic cycle [17]. RTA is an immediate-early gene whose expression is required and sufficient to induce many of the lytic genes and a set of host genes, which collectively are necessary for the induction of viral DNA replication and virus production [17,18,19]. RTA promoter has bivalent chromatin enriched in both the activating H3K4me3 and the repressive H3K27me3 histone marks, which can inhibit RTA expression during latency, while simultaneously priming the RTA promoter for rapid activation during the lytic cycle [6,7]. Several antagonistic epigenetic factors have been reported to be capable of binding to and regulating the activity of the RTA promoter. These include specific histone methyltransferases and demethylases as well as histone acetyltransferases and deacetylases, which can determine whether KSHV remains in latency or undergoes lytic reactivation in latently infected cells [6,10,20,21,22].

There is growing evidence that the chromatin structure of the latent KSHV episome is established in a stepwise manner following *de novo* infection [23,24]. We have previously shown that, during primary infection, the KSHV genome first acquires a transcriptionally permissive chromatin state (euchromatin) favoring the expression of lytic genes, which is then followed by LANA-mediated recruitment of PRC2 and the deposition of H3K27me3 onto the lytic genes after 24 hours post-infection [23,25]. The heterochromatinization of lytic genes ultimately results in the genome-wide silencing of viral lytic genes on the KSHV genome. Interestingly, there is a transient expression of lytic genes in the first 24 hours of infection before PRC2 is recruited and heterochromatin forms on the KSHV genome [23,26]. However, the lack of full-scale lytic gene expression in the first 24 hpi when the viral DNA is devoid of heterochromatin suggests that there must be host restriction factors other than PRC2 that may be recruited to the KSHV genome to suppress lytic gene expression early on during *de novo* KSHV infection. In fact, we have recently shown that cohesin proteins can function as such host restriction factors for the KSHV lytic cycle during primary infection [27]. In addition, we have also demonstrated that while the PRC2-mediated H3K27me3-marked heterochromatin forms on the viral episome only after 24 hpi, the recruitment of the PRC1 factor RYBP already commences during the first hours of infection. However, the role and the mechanism of the recruitment of PRC1 factors during the first hours of infection are still unknown [23,25]. Interestingly, PRC2-mediated H3K27me3 fails to accumulate on the incoming KSHV episome in oral epithelial cells, which can be explained by the fact that oral epithelial cells support lytic infection [23,28]. Thus, the current view is that depending on whether or not heterochromatin forms on lytic genes of the incoming viral DNA can determine if KSHV goes into latency or enters the lytic cycle following infection. However, despite the importance of viral latency in KSHV pathogenesis, we still have limited information about the repertoire of host epigenetic factors that are crucial for the establishment and maintenance of KSHV latency.

To identify host epigenetic factors critical for repressing the lytic genes during KSHV infection that may be important for the establishment and maintenance of viral latency, we performed an siRNA screen by targeting 392 host epigenetic factors during *de novo* KSHV infection. As a result, we identified the Nucleosome Remodeling and Deacetylase (NuRD) complex, Tip60 and Tip60-associated co-repressors, and the histone demethylase KDM2B as inhibitors of the KSHV lytic cycle both during primary infection and in latently infected cells. We found that KDM2B rapidly binds to the viral episome as early as 8 hpi during *de novo* KSHV infection and repress lytic gene expression in the first 24 hours of infection. Our results showed that KDM2B, by binding to the RTA promoter, reduces the levels of H3K4me3 and H3K36me2 activating histone marks on ORF50 at 24 hpi favoring the downregulation of RTA expression, which is necessary for the establishment of viral latency. Taken together, our siRNA screen revealed several novel host epigenetic factors that are involved in the inhibition of lytic genes to facilitate the establishment and maintenance of KSHV latency.

## Materials and methods

### Cell lines and KSHV infection

HEK293T (ATCC), SLK (NIH AIDS Reagent Program), and iSLK cells (obtained from Jae Jung at the University of Southern California) were maintained in DMEM supplemented with 10% fetal bovine serum (FBS) and penicillin-streptomycin (P/S). BCBL1 (NIH AIDS Reagent Program) was cultured in RPMI containing 10% FBS and P/S. Low passage pooled primary human gingival epithelial (HGEP) cells (Zen-Bio) were cultured in CnT-Prime epithelial cell culture medium (Zen-Bio). The iSLK cell line carrying the wild-type KSHV clone BAC16 was cultured in DMEM with 10% FBS, P/S, 250 μg/ml G418, 1 μg/ml puromycin, and 1 mg/ml hygromycin [16,29]. 293TBAC16 wild-type and RTA-KO cell lines were cultured in DMEM with 10% FBS, P/S, and 300 μg/ml hygromycin. TRExBCBL1-3xFLAG-RTA cell line was cultured in RPMI medium containing 10% Tet System Approved FBS (TaKaRa), P/S, and 20 μg/ml hygromycin [19].

### KSHV production, infection, and virus tittering

KSHV reactivation in the TRExBCBL1-3xFLAG-RTA cell line was induced with 1 μg/ml Doxycycline. KSHV BAC16 production and BAC16 infection was performed as described previously [27]. To determine the virus titer in the supernatants derived from shRNA-treated BCBL1 cells we collected the cell culture media after 3 days of lentiviral infection. Virion-associated KSHV DNA in the media was isolated by using DNAzol reagent (ThermoFisher), and the quantity of KSHV DNA was determined by real-time qPCR.

#### Cell viability assay

BCBL1 and HEK293T cells were infected with shRNA lentiviruses for 3 days. The cells were washed in FACS buffer (1% FBS with 4 mM EDTA in PBS) and stained with live/dead discrimination dye (ThermoFisher) according to the manufacturer's protocol. Afterwards, the cells were fixed by 4% paraformaldehyde, washed with PBS, and then resuspended in FACS buffer. The live/dead signals were quantified on an LSR-II or an LSR-Fortessa flow cytometer (BD Biosciences). After removing doublet cells, the flow cytometry data were analyzed by FlowJo.

### Antibodies and oligos

The list of antibodies used for ChIPs and immunoblot analyses, and the sequences of oligos used for qPCR are summarized in S1 and S2 Tables, respectively.

### shRNA lentivirus production and shRNA knockdown

The shRNAs were expressed from a pLKO.1 lentiviral vector. We used pCDHCMV-MCS-EF1puro lentiviral vector to make KDM2B-expressing lentiviruses. In the CXXCm KDM2B mutant cysteine at position 613, 616, and 619 were changed to alanine. The JmjC KDM2B mutant was generated by mutating histidine at position 242 to alanine. The shRNA target sequences are shown in S3 Table. Lentivirus production and the lentiviral infection were performed as described previously [27].

### Total RNA, DNA isolation and their qPCR analysis

The RNA and DNA extraction and qPCR analysis were performed as described in our previous publication [27]. The RT-qPCR primers for KSHV and the host genes are listed in 5’ to 3’ orientation in S2 Table. The RT-qPCR results were calculated as an average of three independent experiments. For significance test, we used a two-tailed student’s t-test where p<0.05 was considered significant.

### Epigenetic factor siRNA screen

SLK cells were first infected with KSHV BAC16 for 2 hours and then the infected cells were washed with 1X DPBS, trypsinized, and seeded onto the siRNA transfection complex in 48-well plates. Reverse transfection was performed with the Lipofectamine RNAiMAX reagent (ThermoFisher) by following the manufacturer’s instruction. We used a custom-designed human siGENOME SMARTpool siRNA library (Dharmacon), which can target 392 different host factors involved in epigenetic regulation (S4 Table). Each host factor was targeted by a mixture of 4 siRNAs. The cells were collected 60 hours after transfection and total RNA was extracted by using the RNeasy kit (Qiagen). Total RNA was used for cDNA synthesis, which was followed by RT-qPCR for RTA, LANA, and 18S genes. The transcript levels of RTA and LANA were normalized with 18S expression and the viral gene expression in the epigenetic factor siRNA-treated cells was calculated relative to the siControl sample using the 2^**-**ΔΔCt^ method to get the relative fold change. For significance test, the two-tailed student’s t-test was performed.

### Immunofluorescence microscopy (IF)

BCBL1 cells were infected with shRNA lentiviruses for 3 days. Afterwards, 2x10^5^ of infected cells were plated onto cover slips pre-coated with Poly-L-Lysine (Sigma) in a 24-well plate and centrifuged for 20 min at 2000 rpm at room temperature. The attached cells were washed with PBS and then subjected to IF analysis. We performed the IF analysis with BCBL1 and the iSLKBAC16-3xFLAG-LANA cell line as described previously [25]. The antibodies and their dilutions used in IF analyses are listed in S1 Table.

#### Chromatin immunoprecipitation (ChIP) assay

The ChIP assay was performed as we have published previously [27]. For the *de novo* infection time-course experiment first SLK cells were infected with shRNA lentiviruses for 2 days followed by KSHV infection for 8, 24, and 72 hours. The ChIP graphs show the average of three independent ChIP experiments, which was calculated as the percentage of the immunoprecipitated DNA compared to input DNA. The ChIP antibodies are listed in S1 Table while the ChIP-qPCR primer sequences are shown in 5’ to 3’ orientation in S2 Table.

## Results

### Identification of novel epigenetic factors critical for the establishment of KSHV latency using a targeted siRNA screen

**Fig 1:**
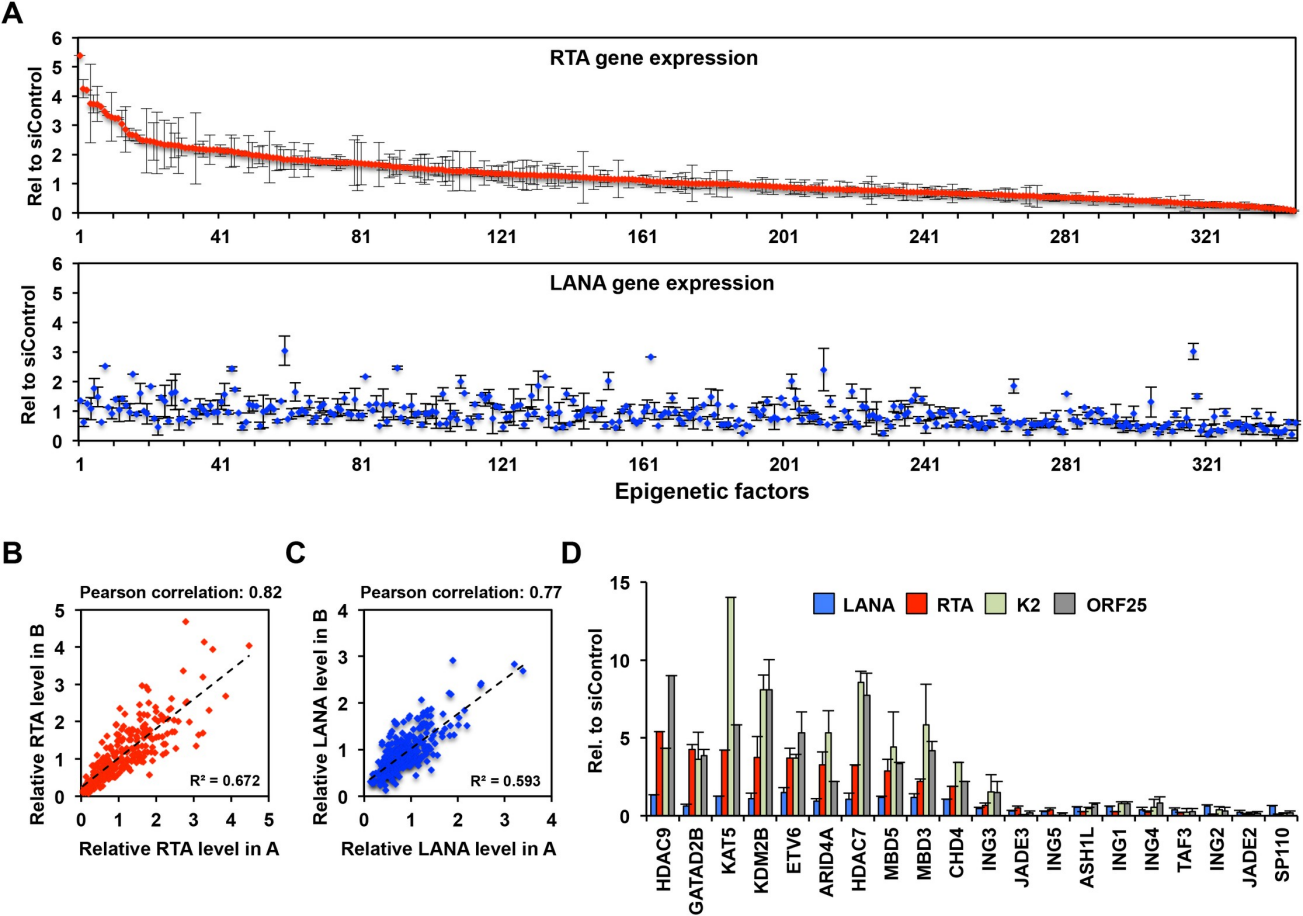
Analysis of viral gene expression changes upon siRNA knockdown of host epigenetic factors during primary KSHV infection of SLK cells. **(A)** RT-qPCR measurement of RTA (top) and LANA (bottom) gene expression. The fold change of viral gene expression in the epigenetic factor siRNA-treated cells was calculated relative to the siControl cells. The average of two biological replicates is shown. Error bars represent standard deviation. The data were sorted and shown based on RTA gene expression. **(B and C)** Comparison of RTA (B) and LANA (C) expression levels in our two independent experimental datasets. Pearson correlation coefficients and R-squared values are indicated. **(D)** Additional RT-qPCR analysis of viral gene expression in a select group of epigenetic factor siRNA-treated samples.

We have previously shown that heterochromatin involved in the inhibition of lytic genes is established on the KSHV genome in SLK cells by 72 hpi [23]. To identify host epigenetic factors that are required for the establishment of KSHV latency, we performed an siRNA screen in SLK cells by targeting 392 host epigenetic factors during *de novo* KSHV infection (Fig 1). We reasoned that if the siRNA knockdown of an epigenetic factor increased lytic gene expression during *de novo* KSHV infection, it would indicate that the epigenetic factor is involved in the inhibition of lytic gene expression and is likely crucial for the establishment of KSHV latency. To avoid the possibility that the siRNA knockdown of epigenetic factors may interfere with viral infection, we first infected SLK cells with KSHV for 2 hours, which was then followed by reverse transfection of the infected cells with siRNAs. At 60 hpi we measured the expression of the lytic gene RTA and the latent gene LANA by RT-qPCR. Fig 1A summarizes the result of the siRNA knockdown of 346 epigenetic factors, which was based on two biological replicates. We calculated the average viral gene expression in the epigenetic factors siRNA-treated cells relative to the non-targeting siRNA control cells. We note that 46 siRNAs were excluded from the analysis because we found that the expression of the housekeeping gene 18S, which was used for the normalization of the RT-qPCR data, was greatly reduced in these samples. Our siRNA screens were highly reproducible, which revealed that the siRNA depletion of 49 host epigenetic factors could increase the expression of RTA by more than 2-fold (Fig 1A–1C and S4 Table). Interestingly, out of these 49 host factors the siRNA inhibition of 3 host factors (CBX7, BAZ2A and DHX9) also resulted in increased LANA expression by more than 2-fold (Fig 1A and S4 Table). These data indicated that the majority of host factors that are involved in the repression of RTA do not repress LANA, which is likely due to the different chromatins associated with RTA and LANA genes.

We selected 10 epigenetic factors from our top hits that are known to have some link to polycomb proteins-mediated gene regulation to further test whose siRNA knockdowns could upregulate RTA expression but would not affect LANA expression (Fig 1D). Importantly, we found that the siRNA inhibition of these host epigenetic factors (HDAC9, GATAD2B, KAT5, KDM2B, ETV6, ARID4A, HDAC7, MBD5, MBD3, and CHD4) increased not only the expression of the IE viral gene RTA but also that of early K2 and late ORF25 lytic genes (Fig 1D). Based on this data, we concluded that these 10 selected epigenetic factors are crucial for the inhibition of the lytic cycle of KSHV following *de novo* infection, thus they are likely important for the establishment of viral latency. Interestingly, 7 of the selected 10 epigenetic factors are linked to two different chromatin regulatory complexes. GATAD2B, CHD4, and MBD3 are major components of the Nucleosome Remodeling and Deacetylase complex (NuRD) while HDAC7, HDAC9, KAT5 (also called Tip60), and ETV6 are constituents of the Tip60 co-repressor complexes (Tip60-R) [30,31]. KDM2B is a histone demethylase, which can reduce the level of activating histone marks such as H3K4me3, H3K36me2, and H3K79me2 at its target genes thereby inhibiting their transcription [32,33,34]. In addition, KDM2B was also implicated in the recruitment of PRC1 to unmethylated CpG islands whereby blocking gene expression [35,36]. MBD5 is a member of the methyl-CpG-binding domain (MBD) family, which interacts with the Polycomb repressive deubiquitinase (PR-DUB), but the function of MBD5 is still poorly characterized [37,38]. ARID4A can function as part of the histone deacetylase co-repressor complex mSin3A, which can regulate the expression of cell cycle- and development-related genes [39]. Based on the diverse functions of KDM2B, NuRD and Tip60-R they can potentially inhibit the lytic cycle of KSHV following *de novo* infection by contributing to the different epigenetic layers of the KSHV epigenome.

In the siRNA screen we also identified 61 host factors whose siRNA inhibition decreased RTA expression at least by 2-fold while 24 of the 61 factors also reduced LANA expression (Fig 1A and 1D, S4 Table). Fig 1D shows a select group of epigenetic factors whose siRNA knockdown greatly reduced the expression of both latent and lytic genes. Interestingly, several of these epigenetic factors are subunits of the histone acetyltransferase complex HBO1 such as JADE1-3 and ING4/5, which has been shown to be able to control the latent replication of KSHV thereby the maintenance of the viral episome in infected cells [40]. Therefore, we envisioned that the siRNA inhibition of these HBO1 factors may reduce the viral copy number in cells thereby affecting viral gene expression. Indeed, we confirmed that the viral DNA level was reduced by 60 hpi upon siRNA knockdown of selected HBO1 factors relative to the siControl sample, which explains the overall reduction in viral gene expression (Fig 1D and S1 Fig). Taken together, our siRNA screen identified several new epigenetic factors regulating the inhibition of KSHV lytic genes, which have not yet been implicated in the establishment of KSHV latency. In addition, we also found a number of host epigenetic factors in our siRNA screen, which can be critical regulators of latent gene expression and/or the maintenance of viral DNA during latency.

### Role of KDM2B, NuRD and Tip60 repressor complexes in the maintenance of KSHV latency

**Fig 2:**
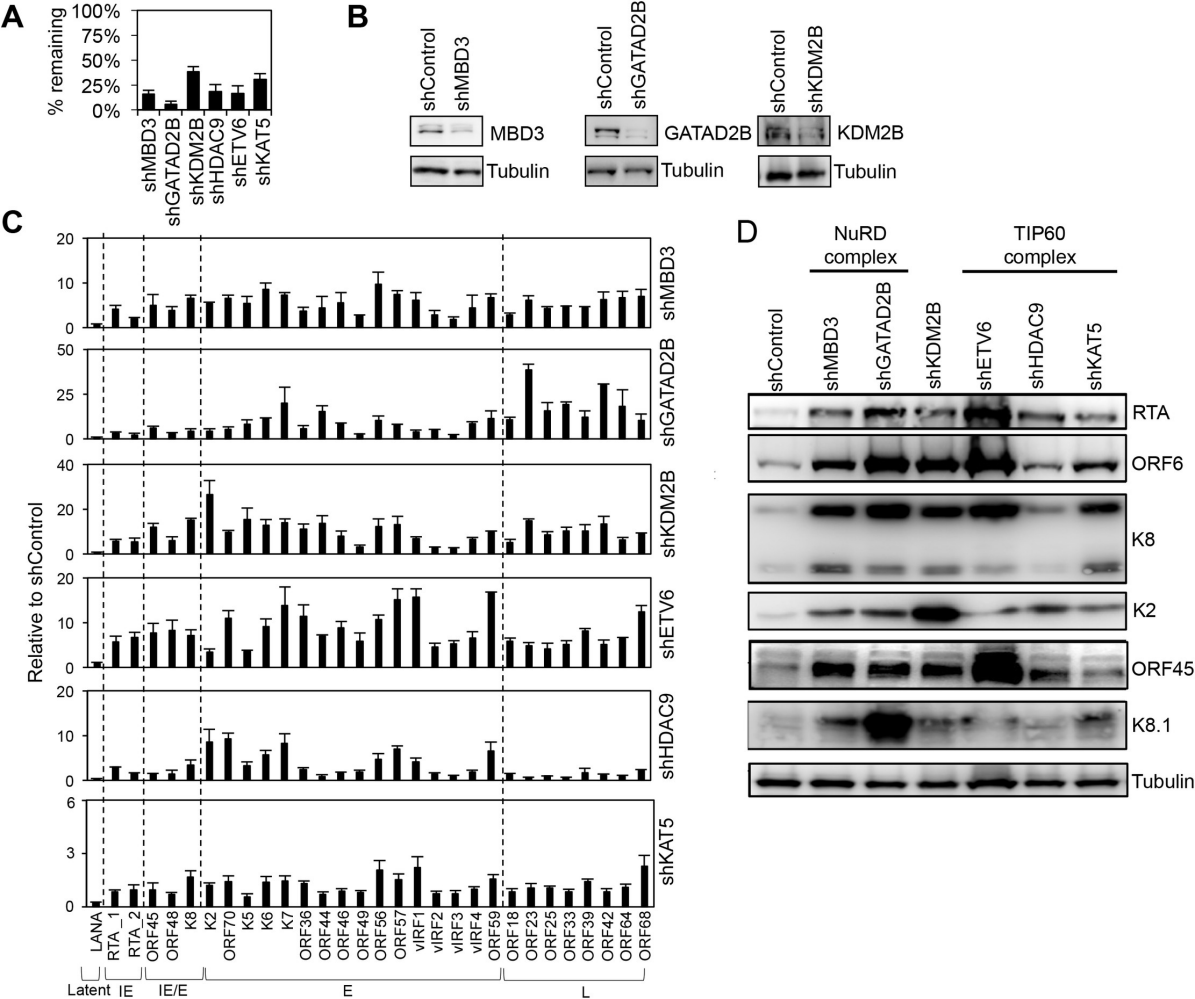
Validation of selected hits by using shRNA-mediated knockdown in latently infected BCBL1 cells. **(A)** RT-qPCR analysis of the expression of epigenetic factors in BCBL1 cells 72 hours after shRNA lentiviral infection. The percentage of remaining transcripts is shown relative to the non-targeting control shRNA (shControl). **(B)** Immunoblot analysis to confirm the shRNA knockdown of MBD3, GATAD2B, and KDM2B at the protein level. **(C)** Effects of the shRNA inhibition of host epigenetic factors on viral gene expression in BCBL1 cells. RT-qPCR was performed for 29 viral genes including latent, immediate early (IE), early (E), and late (L) genes. **(D)** Immunoblot analysis of viral proteins following the shRNA depletion of the host epigenetic factors. Tubulin was used as a loading control.

**Fig 3:**
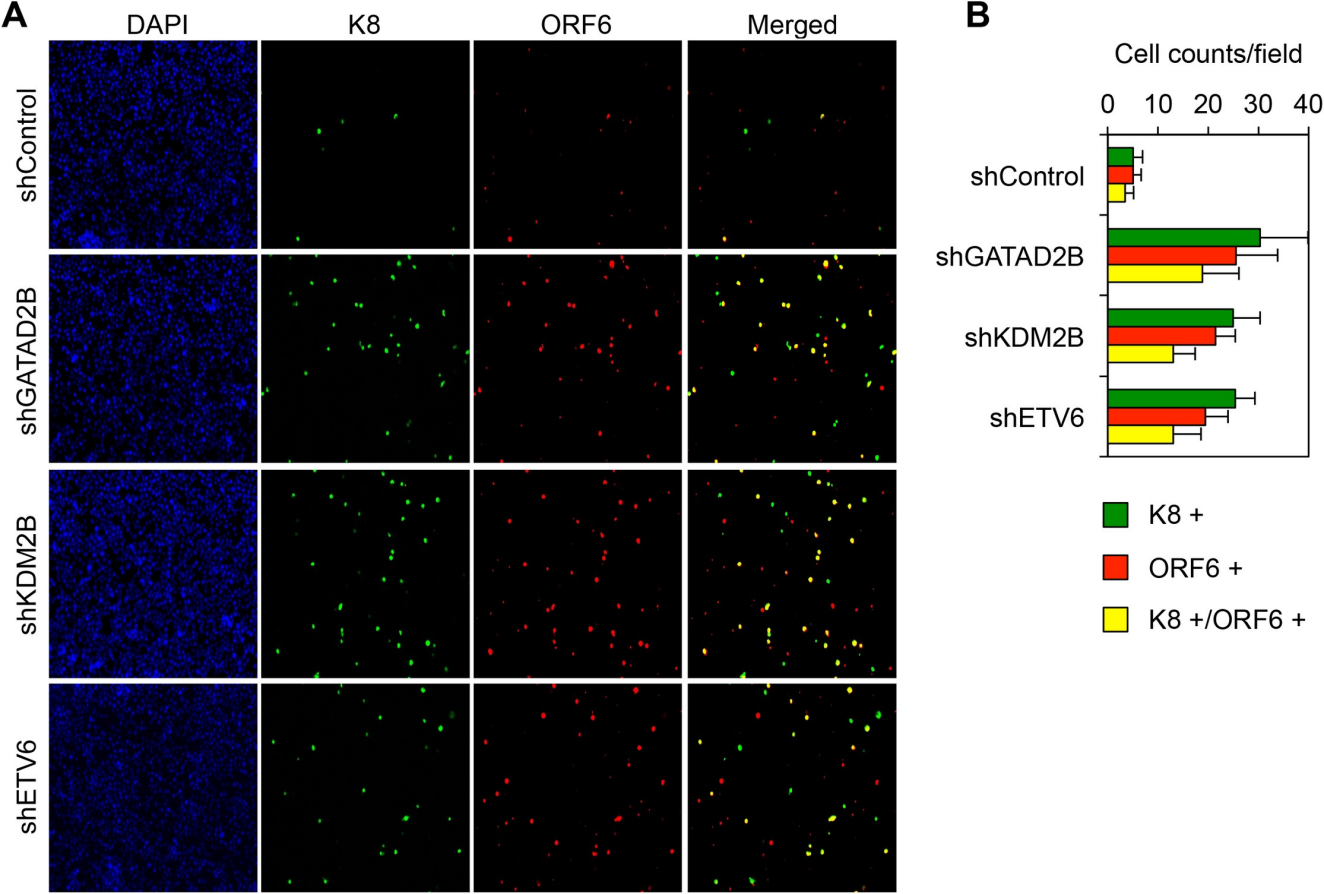
GATAD2B, KDM2B, and ETV6 repress the spontaneous lytic reactivation of KSHV in BCBL1 cells. **(A)** Representative immunofluorescence images showing the expression of ORF6 and K8 lytic proteins in BCBL1 cells 72 hours after shRNA lentivirus infection. **(B)** Quantification of the number of K8, ORF6, and K8/ORF6 positive BCBL1 cells. The average is calculated from at least 10 randomly selected fields.

We and others have shown that host chromatin regulatory factors such as PRCs and the cohesin complex can regulate the KSHV epigenome and viral gene expression during both *de novo* viral infection and in latency [6,7,23,27,41,42]. Therefore, we hypothesized that KDM2B, NuRD, and Tip60-R can also play a role in the inhibition of lytic KSHV genes not only during primary infection but also during latency. To test this idea, we infected the primary effusion lymphoma cell line BCBL1 with lentiviruses, which expressed shRNAs that were specific for MBD3, GATAD2B, KDM2B, HDAC9, ETV6, and Tip60 (Fig 2). Three days after lentivirus infection we analyzed the expression of viral genes at both mRNA and protein levels. Fig 2A and 2B show that we could efficiently reduce the expression of the host epigenetic factors. Strikingly, while none of the epigenetic factor shRNA knockdowns induced LANA expression, all of them could induce lytic gene expression in latently infected BCBL1, which could be detected at both mRNA and protein levels (Fig 2C and 2D). Immunofluorescence analysis also confirmed that the shRNA knockdown of GATAD2B, KDM2B, and ETV6 increased the number of BCBL1 cells expressing lytic viral proteins (K8 and ORF6) (Fig 3). Importantly, the shRNA knockdown of these host epigenetic factors did not affect the viability of cells while they could induce the expression of lytic genes (S2 Fig). These results indicate that KDM2B, NuRD, and Tip60-R are also involved in the repression of lytic genes during latency.

**Fig 4:**
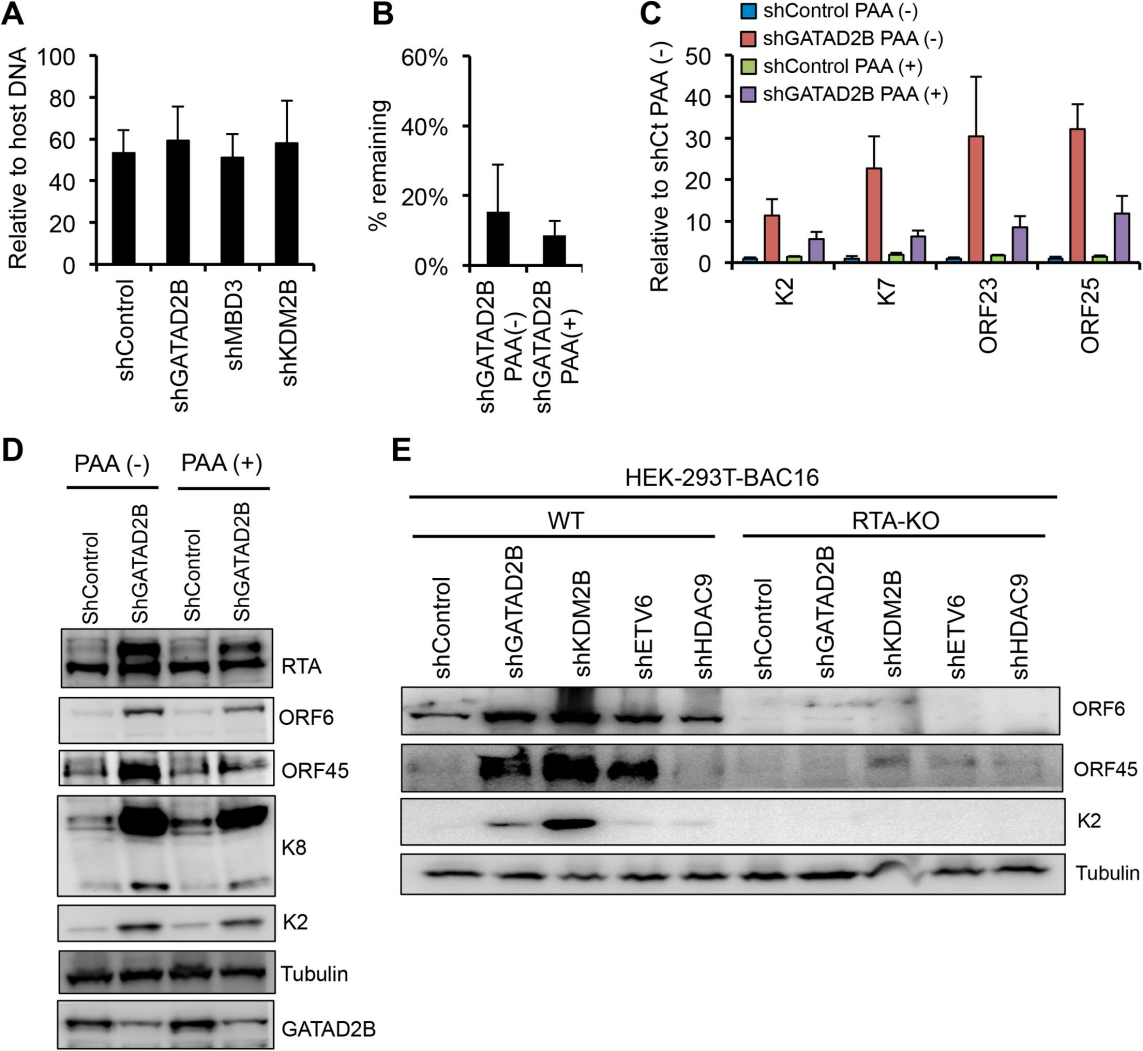
Host epigenetic factors maintain KSHV latency by inhibiting RTA expression and viral DNA replication. **(A)** Viral DNA level in BCBL1 cells following shRNA knockdown of host epigenetic factors. It was measured by qPCR 72 hours after shRNA lentiviral infection. **(B)** Knockdown efficiency of GATAD2B in BCBL1. **(C)** Effect of the viral DNA polymerase inhibitor phosphonoacetic acid (PAA) on the viral gene expression in the GATAD2B depleted BCBL1 cells. **(D)** Immunoblot analysis of viral lytic protein expression in the presence or absence of PAA in shGATAD2B BCBL1 cells. **(E)** Immunoblot detection of lytic viral protein production in 293TBAC16 WT and RTA-KO cells 72 hours after infection with the indicated shRNA lentiviruses. Tubulin was used as a loading control.

The marked increase in the expression late KSHV genes upon the shRNA knockdown of KDM2B, NuRD and Tip60-R factors raised the question if this phenotype could be due to increased lytic viral DNA replication, which is known to be required for robust induction of late viral genes. However, we found that the viral DNA level in shGATAD2B-, shMBD3-, and shKDM2B-treated BCBL1 cells was comparable with the viral DNA level in the shControl cells when the viral DNA load was measured using total cell population (Fig 4A). It is possible that the shRNA knockdown of the epigenetic factors induced viral DNA replication and hence the expression of late genes only in a subset of BCBL1 cells, which cannot be detected by using total cell population. Therefore, to establish if viral DNA replication did occur, which promoted late gene expression that we observed upon the shRNA knockdown of epigenetic factors, we treated BCBL1 cells with PAA during shGATAD2B lentivirus infection (Fig 4B). PAA is a potent viral DNA polymerase inhibitor, which can block lytic viral replication, and consequently also late gene expression. We found that the PAA treatment reduced the expression of not only L genes (ORF23, ORF25) but also that of IE (RTA) and E (K2, K7, ORF6, ORF45, K8) genes in shGATAD2B lentivirus-infected cells (Fig 4C and 4D). In addition, we also measured virus production in BCBL1 cells upon shRNA knockdown of some of the host epigenetic factors. We found that only shKDM2B resulted in slight increase in virus production relative to shControl cells, which was still three orders of magnitude less compared to TPA- or RTA overexpression-induced virus production (S3 Fig). We speculate that the reason we did not see more virus production is that the shRNA knockdown of host epigenetic factors might be incomplete. Alternatively, the shRNA inhibition of epigenetic factors interfere with the induction of RTA’s host target genes that are critical for virus production, which can limit the magnitude of lytic activation (S4 Fig) [19]. Nevertheless, these results suggest that the epigenetic factor shRNA knockdown may also increase lytic viral DNA replication, which can in part contribute to the increased lytic viral gene expression and virus production in a subpopulation of infected cells.

It is known that RTA expression is required for lytic replication and the induction of lytic genes from latency, although some lytic genes can also be induced in a RTA-independent manner outside the canonical lytic cycle [16,18,43]. Therefore, next we wanted to determine whether or not the shRNA inhibition of KDM2B, NuRD or Tip60-R induces lytic genes in a RTA-dependent manner. We infected 293T cells carrying either wild-type (WT) or RTA knockout KSHV in latency with shGATAD2B, shKDM2B, shETV6 and shHDAC9 lentiviruses, respectively (Fig 4E). Three days after lentivirus infection, we analyzed the expression of select lytic viral proteins (ORF6, ORF45, K2) (Fig 4E). The results showed that the production of lytic proteins was largely reduced in the epigenetic factor shRNA-treated cells that carried RTA knockout KSHV. Since RTA is required for downstream lytic gene expression and these epigenetic factors can inhibit RTA expression, we propose based on these results that KDM2B, NuRD, and Tip60-R can inhibit the lytic cycle of KSHV largely through suppressing the expression of RTA.

### Binding of KDM2B to the KSHV episome

**Fig 5:**
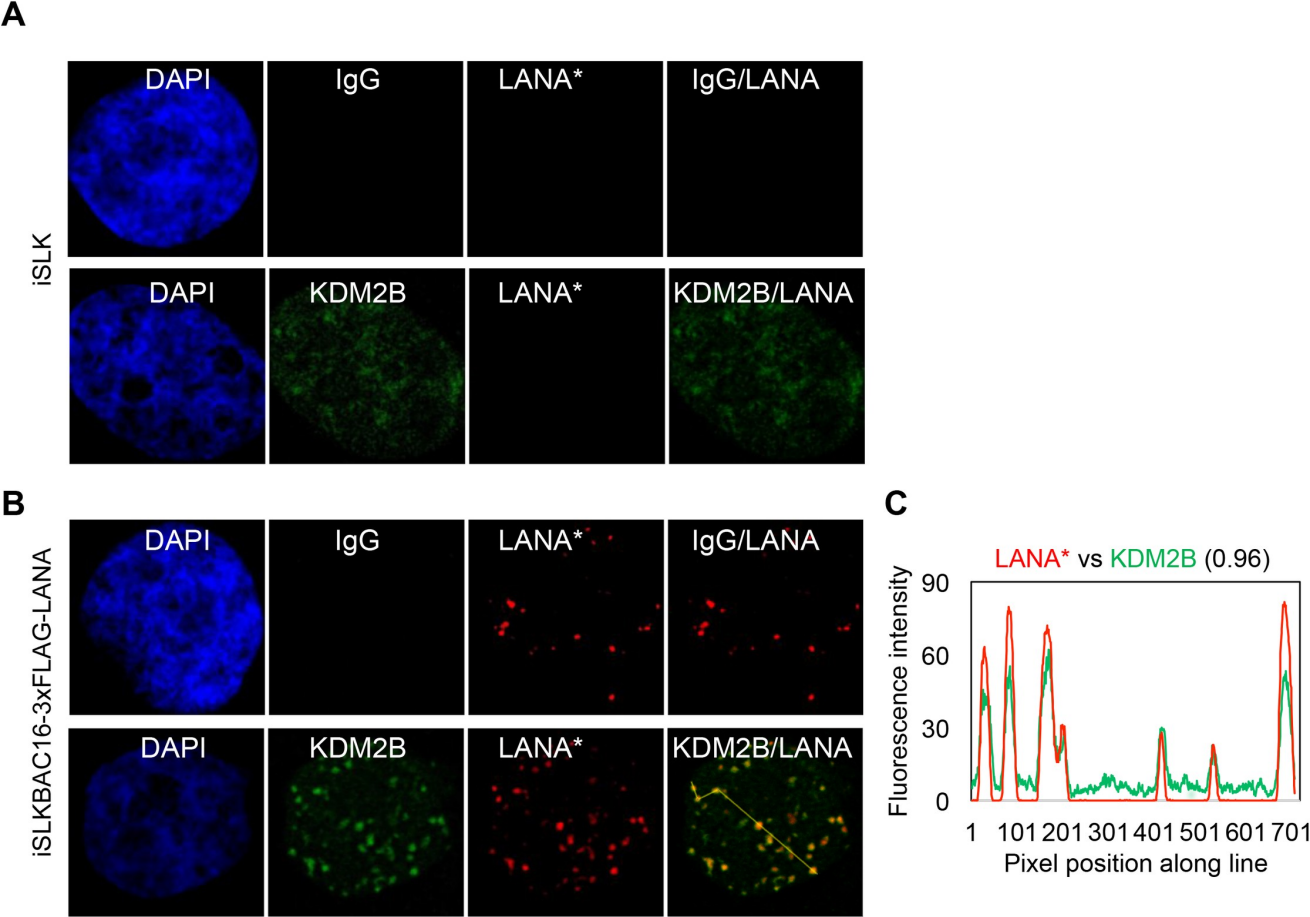
Co-localization of KDM2B with LANA in latent KSHV-infected cells. **(A)** Uninfected iSLK cells or **(B)** KSHV-infected iSLK cells (iSLKBAC16-3xFLAG-LANA) were subjected to immunofluorescence analysis for LANA (red) and KDM2B (green). Rabbit IgG served as a negative control. FLAG antibody was used to detect 3xFLAG-LANA expressed from KSHV. **(C)** Representative LANA puncta were connected by yellow line and the co-localization of LANA with KDM2B along the line was analyzed by ImageJ.

**Fig 6:**
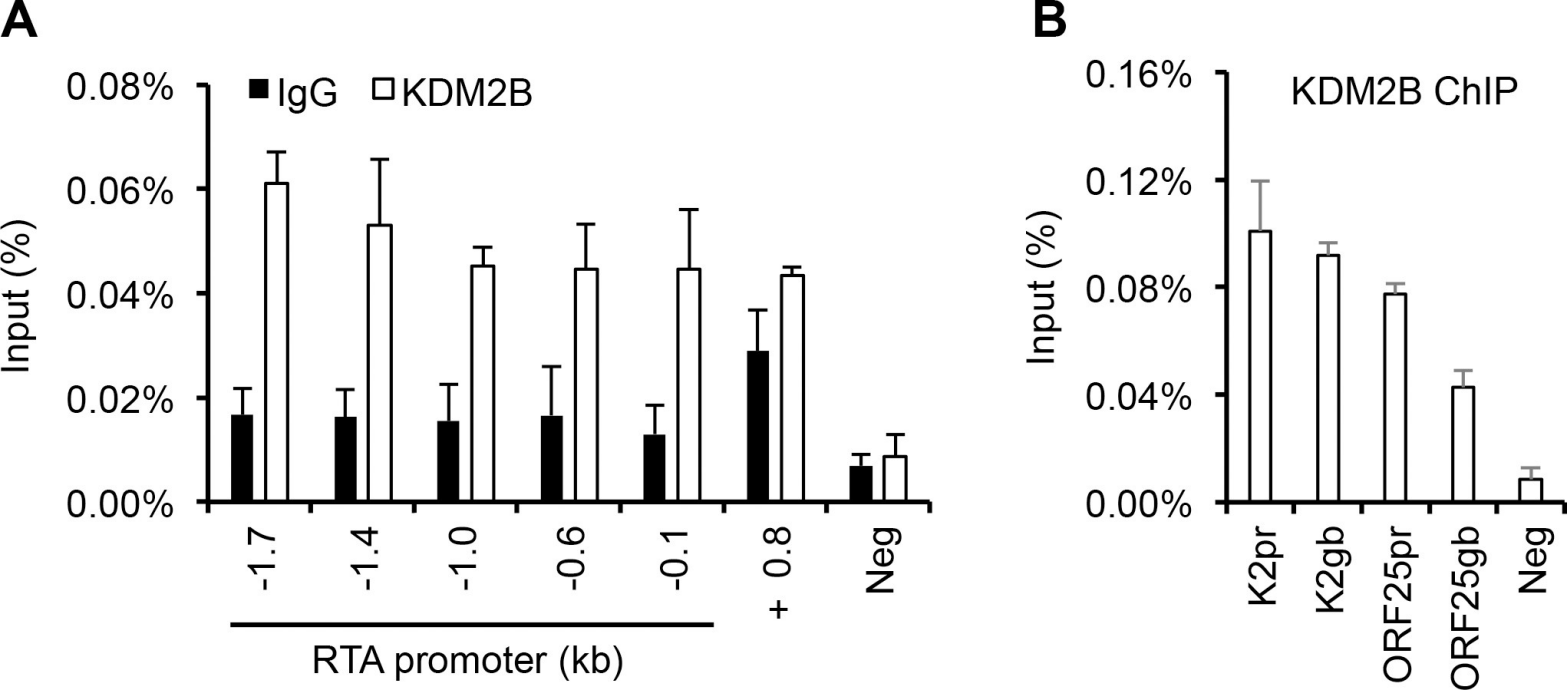
KDM2B binds to the KSHV genome in the latently infected BCBL1 cells. **(A)** KDM2B ChIP assay was performed in latent BCBL1 cells. The KDM2B binding was tested at the indicated regions of RTA promoter and in the RTA gene body +0.8 kb downstream of the transcription start site. IgG ChIP was performed to determine the specificity of KDM2B ChIPs. **(B)** Testing the binding of KDM2B on the promoter (pr) and the gene body (gb) of E (K2) and L (ORF25) genes in latent BCBL1 cells. Cellular intergenic region Neg was used as a negative control.

The inhibitory effect of KDM2B, NuRD and Tip60-R epigenetic factors on lytic genes prompted us to test if they can bind to the KSHV genome thereby directly regulating the expression of lytic genes. To determine whether KDM2B, NuRD or Tip60-R complexes are associated with the KSHV episome in infected cells, we first performed immunofluorescence analyses using a latent iSLK cell line that carries a recombinant KSHV clone BAC16 expressing 3xFLAG-LANA (Fig 5). We relied on LANA to visualize the KSHV episomes in cells, because it is well established that the punctate staining of LANA is due to its binding to the viral episomes in the nuclei of KSHV-infected cells [44]. We investigated the cellular localization of KDM2B, NuRD and Tip60-R factors in uninfected and KSHV-infected cells, and we found that KDM2B was focally enriched and co-localized with LANA in KSHV-infected cells, which impelled us to further investigate the interaction of KDM2B with the KSHV genome (Fig 5 and S5 Fig). Therefore, we performed ChIP experiments using a KDM2B antibody or IgG as a negative control, which revealed that KDM2B could bind to both the promoter and the gene body of IE, E, and L KSHV genes in latent BCBL1 cells (Fig 6). In addition, we found that while KDM2B expression was not decreased, the enrichment of KDM2B was reduced on the RTA promoter in BCBL1 during RTA-induced lytic reactivation (S6 Fig). This was measured at 12 hpi, which represents an early phase of KSHV lytic reactivation prior to lytic viral replication [6]. In conclusion, we provided evidence that KDM2B can bind to the KSHV genome, thus we propose that KDM2B can directly block lytic gene expression.

### KDM2B suppresses lytic gene expression during both *de novo* lytic and latent infections

**Fig 7:**
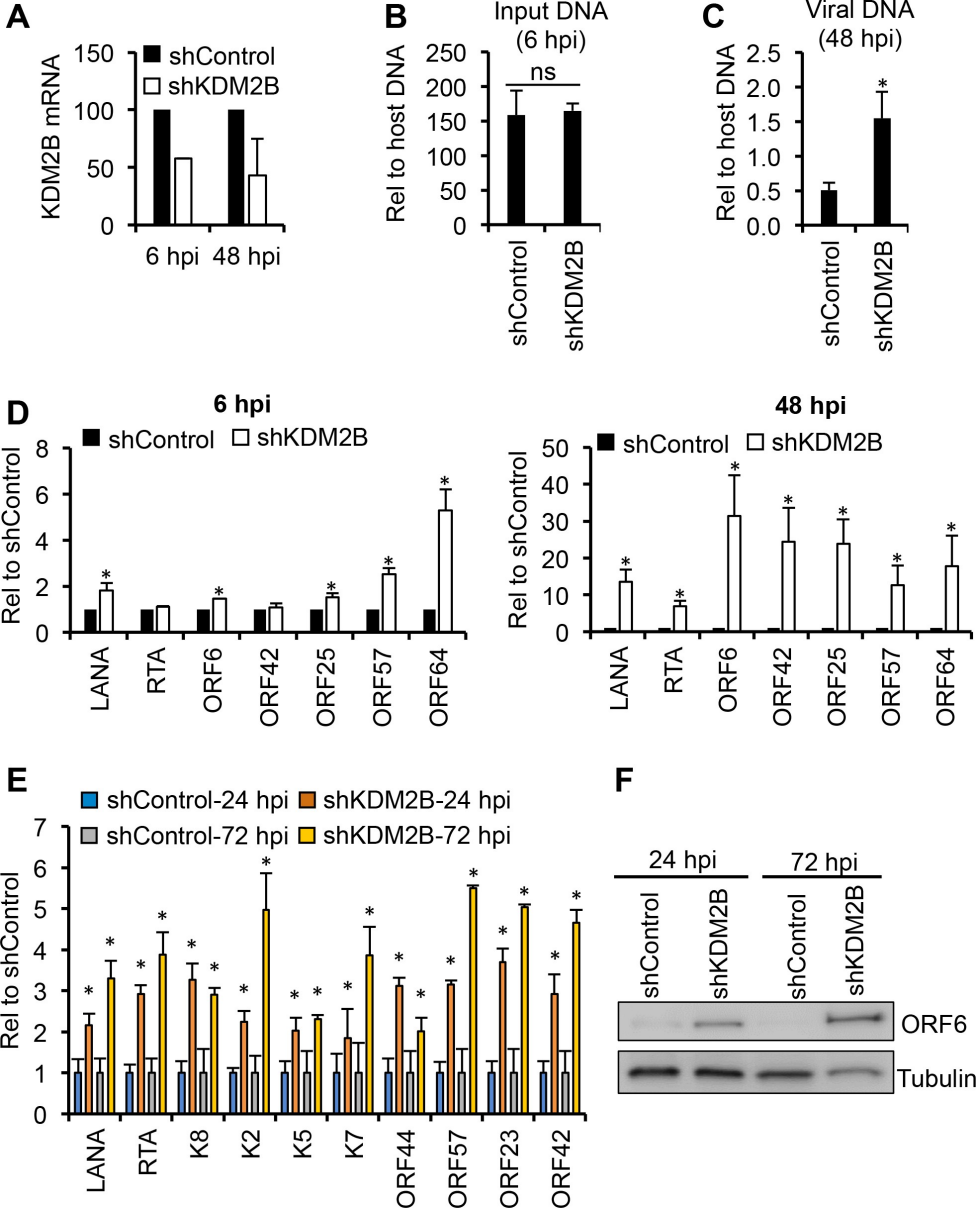
Effect of KDM2B depletion on viral gene expression in different epithelial cell types during *de novo* KSHV infection. **(A)** Knockdown efficiency of KDM2B by shRNA lentivirus infection in primary human gingival epithelial (HGEP) cells. HGEP cells were infected with shControl or shKDM2B lentiviruses for 2 days, followed by KSHV infection. RT-qPCR was performed 6 and 48 hours after KSHV infection to measure viral gene expression. **(B and C)** Viral DNA load in KSHV-infected HGEP cells at 6 hpi and 48 hpi. **(D)** RT-qPCR analysis of viral gene expression. **(E)** RT-qPCR analysis of viral gene expression in shControl and shKDM2B SLK cells 24 and 72 hours after KSHV infection. Note for panel A-E: significance test was performed between shControl and shKDM2B samples (*p<0.05). NS, not significant. **(F)** Immunoblots showing viral ORF6 protein expression at 24 hpi and 72 hpi.

To further test the inhibitory role of KDM2B in lytic gene expression during *de novo* KSHV infection, we infected two different epithelial cell types expressing KDM2B specific shRNA (Fig 7). While primary human gingival epithelial (HGEP) cells support moderate level of lytic viral infection, KSHV establishes latency in the renal carcinoma cell line SLK by 72 hpi following *de novo* infection [23,28]. HGEP cells treated by shControl or shKDM2B were infected with KSHV and then the level of viral DNA and viral gene expression was analyzed at 6 hpi and 48 hpi (Fig 7A–7D). We found that while the input viral DNA load was comparable between shControl- and shKDM2B-treated HGEP cells at 6 hpi (Fig 7B), it was increased by 3-fold in shKDM2B-treated cells compared to shControl-treated cells at 48 hpi (Fig 7C). Strikingly, increased lytic gene expression could be observed in shKDM2B cells compared to shControl cells as early as 6 hpi, which was even more pronounced at 48 hpi when all tested viral genes were upregulated, which is in part likely due to the increased viral copy number (Fig 7D). These data show that KDM2B can also function as a restriction factor of KSHV lytic cycle in primary human oral epithelial cells during the first hours of infection. In agreement with this, and our siRNA screen result, the overall viral gene expression was also increased in *de novo* KSHV-infected SLK cells upon KDM2B shRNA knockdown as early as at 24 hpi, which was further elevated at 72 hpi (Fig 7E and 7F). These results indicate that KDM2B is a rapidly acting inhibitory epigenetic factor, which can impede the lytic cycle of KSHV during both *de novo* lytic and latent infections in different cell types. We also note that, in contrast to EBV, KSHV infection does not reduce KDM2B expression indicating differential gene regulation of KDM2B in EBV- and KSHV-infected cells (S7 Fig and [45]).

### KDM2B rapidly binds to the KSHV genome during *de novo* infection, which requires the DNA-binding domain of KDM2B

**Fig 8:**
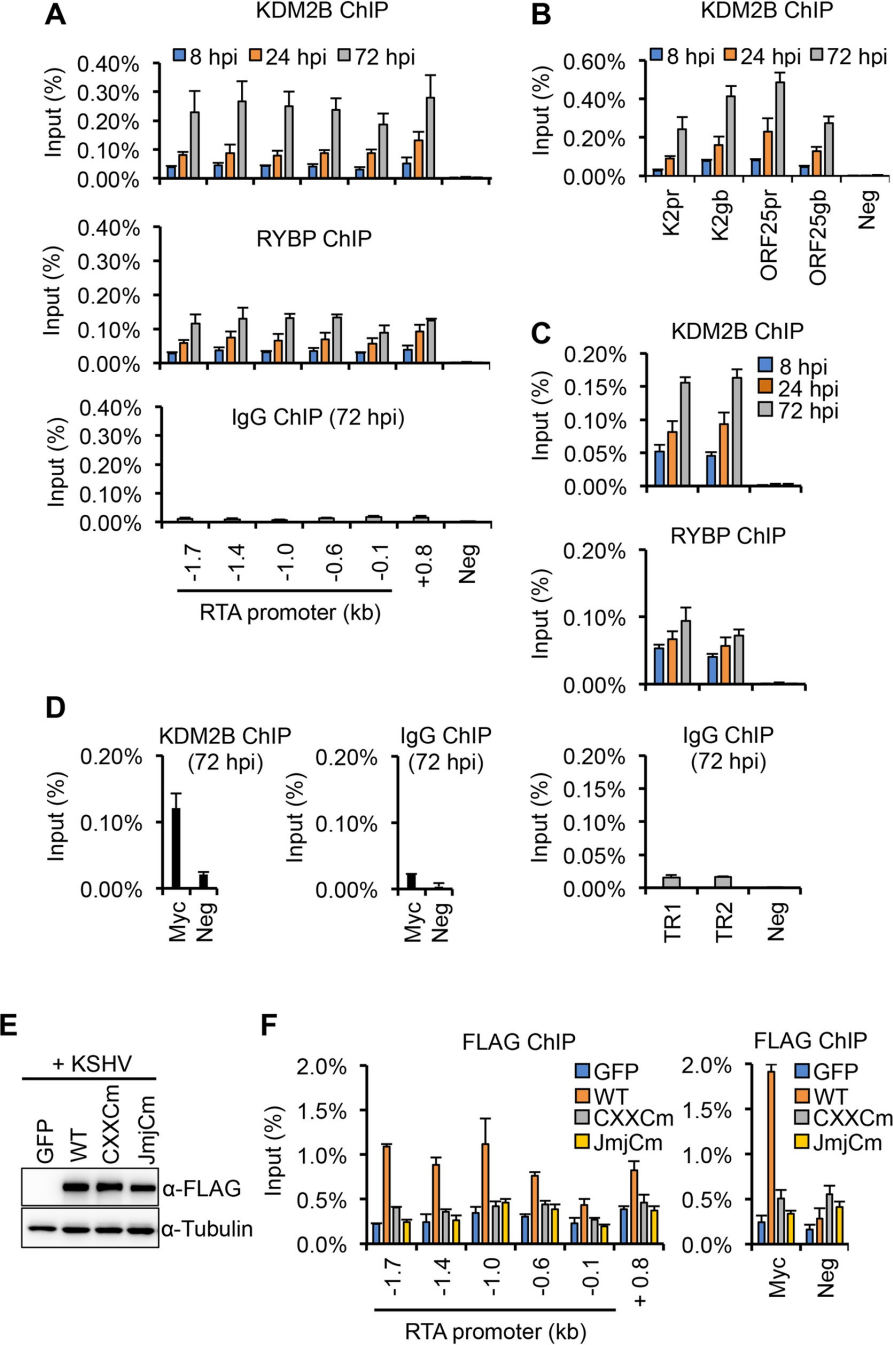
Time course ChIP experiment to test KDM2B-binding on the KSHV genome during *de novo* viral infection. **(A)** SLK cells were infected with KSHV for 8, 24, and 72 hours followed by ChIP for KDM2B, RYBP, and IgG. **(B)** KDM2B-binding on the promoter and gene body of lytic viral genes. **(C)** Binding of KDM2B and RYBP on the terminal repeats (TR) of the viral episome. ChIP-qPCR was performed with two different TR specific primer sets. IgG ChIP was done at 72 hpi. **(D)** KDM2B ChIP on Myc promoter. Cellular intergenic region (Neg) was used as a negative control. **(E and F)** SLK cells were first infected with lentiviruses expressing GFP, WT, CXXCm or JmjCm 3XFLAG-KDM2B for 2 days, which was followed by KSHV infection for 24 hours. Lenti GFP was used as negative control in the experiments. Panel E shows the immunoblot analysis of KDM2B expression using FLAG antibody. Panel F shows FLAG ChIPs to test the binding of 3xFLAG-tagged KDM2B proteins on the RTA promoter and gene body as well as at the promoter of Myc gene.

The fact that KDM2B can bind to the viral episome in latency (Figs 5 and 6) and can inhibit lytic gene expression a few hours after KSHV infection (Fig 7) suggests that KDM2B may rapidly bind to the viral genome during *de novo* infection. To test this idea, we performed a time course KDM2B ChIP assay whereby we could analyze the binding of KDM2B on different parts of the KSHV genome in infected SLK cells at 8, 24, and 72 hpi (Fig 8A–8C). At the same time, we also performed a ChIP for the PRC1 factor RYBP as a positive control, since its binding can be detected on the KSHV genome as early as 8 hpi [23]. In addition, ChIP with IgG was used as a negative control to determine the background level of ChIPs while an intergenic region on the host genome (Neg) was used as an additional negative control for the ChIP assays. As a positive control for the KDM2B ChIP we used Myc promoter where KDM2B is known to bind (Fig 8D) [46]. Our results showed that the enrichment of KDM2B on the RTA promoter was gradually increased along with RYBP while the ChIP signal in the IgG ChIP samples and on the Neg region remained low indicating the specificity of KDM2B and RYBP ChIPs (Fig 8A). Strikingly, the rapid and gradual recruitment of KDM2B was not limited to the RTA promoter but it could also be detected both on the promoter and the gene body of the E gene K2 and the L gene ORF25 (Fig 8B) as well as on the terminal repeats of the viral episome (TR) (Fig 8C). Importantly, the binding of KDM2B to the viral episome at 8 hpi is consistent with its early inhibitory function at viral genes during *de novo* infection.

To investigate whether or not the DNA-binding or the histone demethylase domain of KDM2B is required for its binding to the KSHV genome, we infected SLK cells expressing 3xFLAG-tagged WT, a DNA-binding (CXXCm) or a histone demethylase domain mutant (JmjCm) of KDM2B with KSHV [36]. Immunoblot analysis showed that the expression of WT KDM2B and its mutants was comparable (Fig 8E). At 24 hpi we analyzed the binding of FLAG-tagged KDM2B to the promoter of RTA by using FLAG ChIP assay (Fig 8F). We found that while WT 3xFLAG-KDM2B could efficiently bind to the RTA promoter, the binding of the KDM2B mutants was largely abrogated indicating that both the DNA-binding and the histone demethylase domains of KDM2B are required for being able to bind to the viral genome (Fig 8F).

### Recruitment of KDM2B onto the RTA promoter is independent of LANA

**Fig 9:**
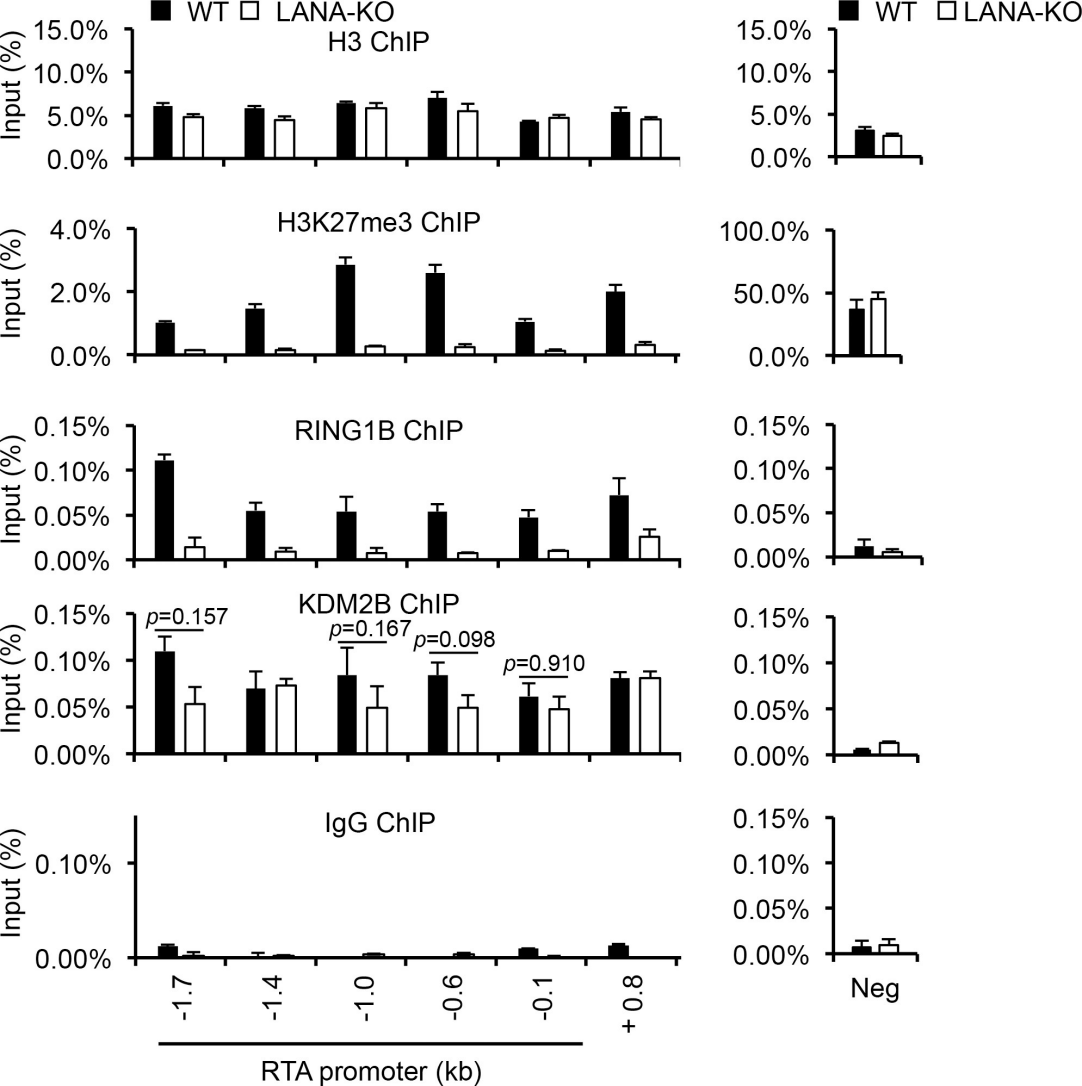
KDM2B-binding on the KSHV genome is independent of LANA. SLK cells were infected with WT or LANA-KO (knockout) KSHV for 72 hours and then ChIPs were performed. Rabbit IgG ChIP was used as a negative control. The cellular intergenic region Neg was used as control for the ChIPs.

We have previously shown that the recruitment of PRC2, the deposition of H3K27me3, and the H3K27me3-promoted enrichment of PRC1 on the KSHV genome by 72 hpi depend on LANA during *de novo* viral infection [25]. Since both KDM2B and LANA can bind to the viral episome within hours of infection, we wanted to determine if LANA is involved in the recruitment of KDM2B to the viral DNA. For this, we used KDM2B ChIP to compare KDM2B-binding on the RTA promoter in WT and LANA knockout (LANA-KO) KSHV-infected SLK cells at 72 hpi. Fig 9 shows that while both the H3K27me3 enrichment and the binding of the PRC1 factor RING1B were abolished on the entire RTA promoter in LANA-KO KSHV-infected cells, histone H3 and KDM2B-binding was largely unaffected, and only slightly reduced at a few sites of the RTA promoter. These ChIP experiments indicated that in contrast to LANA-mediated recruitment of PRCs-regulated heterochromatin, LANA is largely dispensable for the recruitment of KDM2B to the RTA promoter during *de novo* KSHV infection.

### KDM2B reduces the level of activating histone marks on the RTA promoter during *de novo* KSHV infection

**Fig 10:**
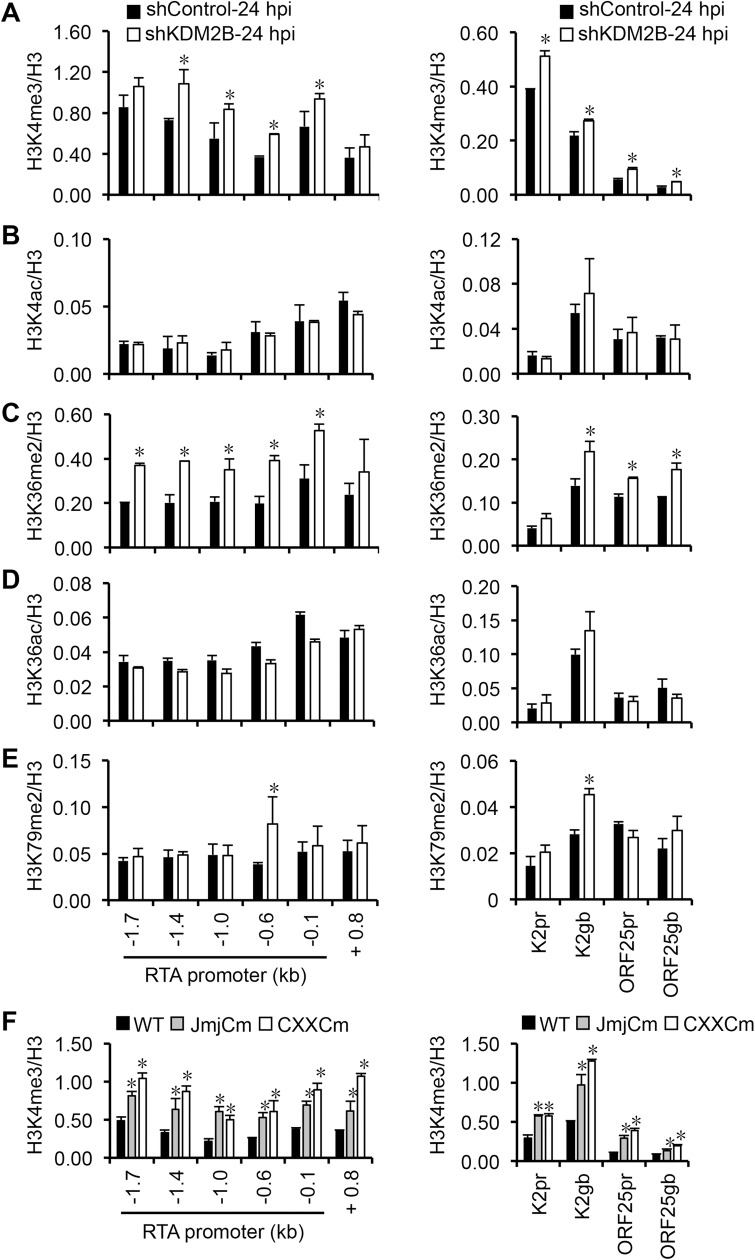
Effect of KDM2B on the level of activating histone marks on the KSHV genome. SLK cells were infected with shControl and shKDM2B lentiviruses for 48 hours followed by KSHV infection for 24 hours. ChIP was performed for **(A)** H3K4me3, **(B)** H3K4ac, **(C)** H3K36me2, **(D)** H3K36ac, and **(E)** H3K79me2. The graphs show the histone mark ChIP signals relative to the level of histone H3 at each genomic region. Significance test was performed between shControl and shKDM2B samples (*p<0.05). **(F)** Effect of the overexpressed WT, JmjCm, and CXXCm KDM2B on the level of H3K4me3 on the viral genome. The SLK cell line was infected with lentiviruses expressing 3XFLAG-tagged WT or mutant KDM2B for 2 days followed by KSHV infection for 24 hours. H3K4me3 and H3 ChIPs were performed and the ChIP signal is shown relative to the level of histone H3. Error bars represent standard deviation (n = 3). Significance test was performed between WT and mutant KDM2B expressing samples (*p<0.05).

KDM2B is a histone demethylase, which has been shown to be able to reduce the level of H3K4me3, H3K36me2, and H3K79me2 activating histone marks at specific target genes thereby controlling the expression of specific host genes [32,33,34]. In addition, KDM2B can also recruit PRC1.1 that includes RING1B, the E3 monoubiquitin ligase of H2AK119, to CpG islands in a PRC2-independent manner [35,36]. To test if KDM2B can regulate the enrichment of any of the activating histone marks on the viral episome during *de novo* KSHV infection, we performed histone mark ChIPs on the RTA, K2, and ORF25 viral genes in shKDM2B-treated SLK cells following 24 hours post-infection (Fig 10). Because H3K4 and H3K36 can be either methylated or acetylated, we tested for not only H3K4me3, H3K36me2, and H3K79me2, but also H3K4ac and H3K36ac to determine the specificity of KDM2B activity on the viral genome (Fig 10A–10E). Our ChIP analysis showed that the shRNA knockdown of KDM2B resulted in a significant increase of H3K4me3 and H3K36me2 levels whereas the acetylation of H3K4 and H3K36 were not significantly affected on the RTA promoter during *de novo* KSHV infection. Similar histone mark changes could also be observed at K2 and ORF25 but it was less pronounced compared to RTA. Interestingly, while the level of H3K79me2 was comparable at most of the genomic regions tested on the viral episome in shControl and shKDM2B cells, it did increase in K2 gene body (K2 gb) and at RTA-0.6 kb region, which corresponds to not only RTA promoter but also ORF48 gene body. These results indicate that KDM2B can reduce the level of activating histone marks on lytic genes during *de novo* KSHV infection. In contrast, while shKDM2B resulted in reduced binding of RING1B on the Myc promoter, which is a known target of KDM2B, we could not detect any significant changes in RING1B enrichment in most parts of the RTA locus during KSHV *de novo* infection (S8 Fig). These results indicate that KDM2B can control the enrichment of activating histone marks but it may not play a major role in the recruitment of PRC1 to the viral episome. Alternatively, the shRNA-mediated depletion of KDM2B might not be sufficient enough to see its effect on PRC1 recruitment.

To further demonstrate that KDM2B can control the level of activating histone marks on the RTA promoter, we also tested whether the overexpression of KDM2B affects the level of H3K4me3 on the KSHV genome during primary infection. To this end, SLK cells expressing either WT or the histone demethylase mutant (JmjCm) or the DNA-binding mutant of KDM2B (CXXCm) were infected with KSHV, and then H3K4me3 ChIP was performed on the RTA promoter at 24 hpi (Fig 10F). We found that the level of H3K4me3 was reduced on the RTA promoter in WT KDM2B-overexpressed SLK cells compared to the JmjCm and CXXCm KDM2B-overexpressed cells (Fig 10F). Importantly, this result is in agreement with the KDM2B mutants being unable to bind to the RTA promoter while WT KDM2B can do so (Fig 8E) thereby the overexpressed WT KDM2B can target RTA promoter and reduce the level of H3K4me3 on it. In summary, our results indicate that KDM2B rapidly binds to the KSHV genome and reduces the level of activating histone marks at the promoter of lytic genes during *de novo* viral infection, which can contribute to the KDM2B-mediated inhibition of lytic genes thereby promoting the establishment and maintenance of KSHV latency.

## Discussion

Establishment of viral latency is an essential step in herpesvirus infection, which facilitates the persistent infection of their host. The first step to latency involves the genome-wide inhibition of lytic genes following *de novo* viral infection, which is still a poorly understood process. Thus, the goal of this study was to reveal the repertoire of host epigenetic factors, which can suppress lytic gene expression following primary KSHV infection denoting their crucial role in the establishment of viral latency. Using an epigenetic factor siRNA screen during primary infection, we identified several epigenetic factors that can inhibit the expression of RTA thereby potentially contributing to the establishment of latency. Of these host factors, we showed that NuRD, Tip60 and its co-repressors, and KDM2B could inhibit lytic genes both following primary infection and during latency in different cell types. Since we found that KDM2B was focally enriched in latent KSHV-infected cells and significantly co-localized with the viral episome, we further dissected the role of KDM2B in KSHV infection. Collectively, our results show that KDM2B inhibits the lytic cycle of KSHV following *de novo* infection mainly by binding to the RTA promoter as early as 8 hpi and reducing the level of specific activating histone marks on it at 24 hpi. Importantly, the removal of activating histone marks, especially H3K4me3, by KDM2B can favor the downregulation of RTA expression during primary infection, which is required for the establishment of viral latency. Importantly, NuRD, Tip60, and KDM2B have also been shown to be able to control other viral infections and viral pathogenesis such as in the case of human papillomavirus, human cytomegalovirus, Epstein-Barr virus, arbovirus, and influenza virus infections [45,47,48,49,50,51]. Thus, the characterization of the role of NuRD, Tip60, and KDM2B factors in viral infections can help better understanding of different viral pathogeneses.

The fact that we were able to identify several subunits of larger multiprotein complexes such as the NuRD complex and a number of Tip60-associated co-repressors as RTA inhibitors in our siRNA screen signifies the specificity of our assay. The NuRD complex is composed of a set of proteins with diverse functions such as ATP-dependent chromatin remodeling, histone deacetylation, and DNA- and histone-binding [30]. Host genes regulated by NuRD include those involved in DNA repair, cell cycle regulation, development, and aging [52]. We found that the siRNA knockdown of the ATPase chromatin remodeler CHD4, the CpG island-binding factor MBD3, and the DNA-binding factor GATAD2B, which can potentiate NuRD-mediated transcription repression, resulted in increased lytic gene expression in KSHV-infected cells. While NuRD is known to be a potent transcription silencer, it was surprising to see Tip60 as a transcription inhibitor for lytic KSHV genes. Tip60 was originally isolated as a HIV-1 Tat-interactive protein, 60 kDa, which functions as a histone acetyltransferase [53,54]. Tip60 plays a role in the regulation of DNA damage response, apoptosis, and cell cycle [55,56]. Interestingly, Tip60 can act either as a transcriptional co-activator or co-repressor, depending on its interaction partners. When Tip60 forms a complex with HDAC7, HDAC9 or ETV6, these Tip60 repressor (Tip60-R) complexes can act as transcription inhibitors [57,58]. Moreover, Tip60-mediated H4K12ac histone mark has also been linked to gene repression. H4K12ac, which is highly enriched in pericentric heterochromatin, can serve as a binding site for epigenetic repressors [59]. While Tip60 has been previously found to directly interact with LANA, the role of neither Tip60 nor NuRD in the regulation of lytic genes has been studied during *de novo* KSHV infection [60]. We note that a previous study by Simpson et al. showed that Tip60 promotes lytic KSHV gene expression, which differs from our conclusion [61]. We used Tip60 shRNA both during primary KSHV infection of SLK cells and in latently infected BCBL1 cells to show that Tip60 is involved in the repression of KSHV lytic genes. In contrast, Simpson et al. performed Tip60 shRNA knockdown in HEK293T cells carrying BAC36 and used histone acetyltransferase inhibitors, which have pleiotropic effects in BCBL1 [61]. Thus, we think that the difference between our results can be due to using different cell lines, KSHV clones, and methodology for testing the impact of Tip60 on KSHV lytic genes. Because we found that in addition to Tip60 several of the known co-repressors of Tip60 were also recovered in our siRNA screen, our findings suggest that Tip60-Rs are in fact bona fide repressors of KSHV lytic genes during infection. Since both NuRD and Tip60-R complexes include histone deacetylases, and the level of histone acetylation at lytic genes can greatly affect KSHV gene expression [10], we hypothesize that NuRD and Tip60-R can inhibit lytic gene transcription by reducing histone acetylation in the viral chromatin. However, given that histones can be acetylated at several lysine residues, further studies are required to determine what histone acetylation marks are controlled by NuRD and Tip60-R to inhibit lytic gene expression [62].

Besides histone acetylation, the methylation of histones at specific residues can also play a crucial role in KSHV gene regulation [4,63]. We have previously shown that while the enrichment of H3K4me3 is associated with lytic gene expression, PRC2-mediated H3K27me3 is linked to viral gene repression [6,7,23]. Interestingly, the chromatin of lytic genes is first enriched in H3K4me3 genome-wide in the first 24 hours of infection, which is then followed by the establishment of H3K27me3-marked heterochromatin by 72 hpi during *de novo* KSHV infection [23,24,25]. In this context we identified the histone demethylase KDM2B as an inhibitor of lytic genes, which can rapidly bind to the viral episome during *de novo* KSHV infection and reduce the level of activating histone marks H3K4me3, H3K36me2, and H3K79me2 at lytic genes favoring downregulation of lytic genes. Both H3K4me3 and H3K36me2 have been shown to be able to directly inhibit the activity of PRC2, while Dot1L-mediated H3K79me2 facilitates transcription elongation [64,65]. Thus, we propose that KDM2B is a crucial host restriction factor that limits the level of activating histone marks on the viral chromatin during *de novo* infection to block full-scale lytic gene expression in the early phase of infection when heterochromatin is not yet established on the lytic genes. This is in agreement with our findings that shKDM2B increased lytic gene expression during both latent (in BCBL1 and SLK cells) and lytic infections (in HGEP cells). Recently, KDM2B has also been reported to affect EBV gene expression [45]. However, in contrast to KSHV infection, EBV induces rapid downregulation of KDM2B expression in B cells following primary infection, which is accompanied by a transient enrichment of KDM2B on an EBV gene promoter at 48 hpi. Consequently, the mechanism of KDM2B-mediated viral gene repression may differ between KSHV and EBV, which needs further investigation. Nevertheless, our data along with the effect on EBV infection show that KDM2B is a critical restriction epigenetic factor of gammaherpesvirus infections.

We found that the zinc finger DNA-binding domain of KDM2B is required for its binding to the RTA promoter, which is also needed for reducing H3K4me3 on the RTA promoter at 24 hpi indicating that KDM2B directly affects the viral chromatin. KDM2B has been demonstrated to bind to unmethylated CpG islands on DNA, and since KSHV DNA is largely unmethylated during *de novo* infection, the viral genome is an excellent target for KDM2B [7]. In fact, Gunther T. et al. has recently shown the genome-wide enrichment of KDM2B on the KSHV episome by using ChIP-seq analysis, which is in agreement with our KDM2B ChIP-qPCR data [66]. Recent reports have also shown that KDM2B can recruit the non-canonical form of PRC1 (PRC1.1) to unmethylated CpG islands besides the canonic PRC1 recruitment pathway, which requires PRC2-mediated pre-deposition of H3K27me3 as a docking site for PRC1 [33,36,67]. We could also observe the binding of the PRC1 factor RYBP to lytic KSHV promoters before 24 hpi prior to PRC2 recruitment as shown in Fig 8, which is in agreement with our previous reports [23,25]. However, our previous studies also showed that the inhibition of H3K27me3 deposition on the viral chromatin could significantly reduce PRC1 accumulation by 72hpi [23,25]. Thus, while the bulk of PRC1-binding requires the pre-deposition of H3K27me3 on the viral chromatin, certain PRC1 factors could also be recruited to the KSHV genome in a PRC2-independent manner during the first day of infection. This non-canonical PRC1 recruitment can be mediated by transcription factors or epigenetic factors like KDM2B that can rapidly bind to the KSHV DNA during *de novo* infection [35,36,68]. We note, however, that while KDM2B can still bind to the RTA promoter in LANA-KO KSHV-infected SLK cells, H3K27me3 enrichment and the binding of RING1B, which is a component of all PRC1 complexes, were greatly reduced confirming our previous study (Fig 9) [25]. Thus, despite the binding of KDM2B to the RTA locus RING1B-binding is abrogated, which can be due to the reduced level of H3K27me3 on the RTA promoter as we reported previously [23]. In addition, our ChIP analysis showed that the binding of RING1B was not affected in most parts of the RTA locus in shKDM2B-treated KSHV-infected cells (S8 Fig). Thus, our results suggest that KDM2B may not play a major role in recruiting PRC1 to the viral genome. However, this does not exclude the possibility that there are also other factors besides PRC2, which can contribute to the recruitment of PRC1 onto the KSHV genome. The fact that many of the epigenetic factors that we identified as inhibitors of RTA (e.g. NuRD, ETV6, MBD5, KDM2B) interact directly or functionally with polycomb proteins further highlights the critical role of polycomb proteins and their interaction network in the repression of lytic genes and the establishment and maintenance of viral latency [37,69,70].

NuRD, Tip60-R, and KDM2B with their diverse functions in chromatin regulation can have differential contribution and/or redundant function in the repression of lytic genes, which can explain why their shRNA knockdown resulted in only modest upregulation of distinct lytic genes in KSHV-infected cells (Fig 2). However, because we saw the induction of viral genes from each viral lytic gene expression class (immediate early, early, and late), this is compelling evidence for the role of NuRD, Tip60-R, and KDM2B in genome-wide inhibition of lytic genes in latently infected cells. Fig 4E showed that the shRNA knockdown of these epigenetic factors could not induce robust lytic gene expression in RTA knockout KSHV-infected cells. Thus, we propose that these epigenetic factors block KSHV lytic cycle through inhibiting the RTA promoter thereby suppressing RTA expression, the very first step of the lytic cycle.

We propose that NuRD, Tip60-R, KDM2B along with cohesin and the Polycomb protein complexes contribute to different epigenetic layers of the viral episome, which can all contribute to the establishment, maturation, and the maintenance of the viral heterochromatin in infected cells [6,23,27,41,42]. Disruption of any of these epigenetic layers can result in various level of lytic gene induction, which can elicit immune response detrimental for the KSHV-infected cells. Further studies are warranted to dissect which of the newly identified epigenetic factors control KSHV chromatin directly and which host epigenetic factors affect KSHV gene expression indirectly through regulating host genes in order to enforce viral latency. Since epigenetic processes are highly reversible, we believe that the identification and characterization of epigenetic factors that control the establishment and maintenance of KSHV latency can provide potential therapeutic approaches to stall persistent infection of humans by KSHV.

## Supporting information

S1 Fig Measurement of viral DNA levels in siRNA-treated infected cells. The viral DNA level was tested by qPCR at 60 hpi and calculated relative to the siControl sample. The host factors targeted by siRNAs are indicated along the x-axis.
(TIF)

S2 Fig Analysis of cell viability. **(A)** Testing cell viability in latently infected BCBL1 cells following shRNA knockdown of host epigenetic factors. **(B)** Testing cell viability in HEK293T cells after shRNA knockdown of host epigenetic factors.
(TIF)

S3 Fig Effect of shRNA knockdown of host epigenetic factors on infectious virus production from latently infected BCBL1 cells. BCBL1 cells were infected with shRNA lentiviruses targeting the host epigenetic factors for 3 days and the viral DNA was isolated from the supernatant. The viral DNA was quantified by qPCR and the infectious virus particles were calculated.
(TIF)

S4 Fig Analyzing the effect of shRNA knockdown of host epigenetic factors on RTA-induced host-target genes. BCBL1 cells were infected with shRNA lentiviruses targeting GATAD2B or KDM2B for 3 days. The expression of host genes was analyzed by RT-qPCR and the fold change in gene expression was calculated relative to the shControl-treated sample (ns: not significant, asterisk indicates p<0.05).
(TIF)

S5 Fig Testing the co-localization of host epigenetic factors with LANA in latent KSHV-infected cells. **(A)** Uninfected iSLK cells or KSHV-infected iSLK cells (iSLKBAC16-3xFLAG-LANA) were subjected to immunofluorescence analysis for LANA (red) and GATAD2B or MBD3 (green). **(B)** KSHV-infected iSLK cells (iSLKBAC16-3xFLAG-LANA) were subjected to immunofluorescence analysis for LANA (red) and CHD4 or ETV6 (green). FLAG antibody was used to detect 3xFLAG-LANA expressed from KSHV BAC16.
(TIF)

S6 Fig Analysis of KDM2B-binding on the KSHV genome during latency and lytic reactivation. TRExBCBL1-3xFLAG-RTA cells were treated with 1 μg/ml doxycycline to induce the 3xFLAG-RTA transgene, which results in lytic reactivation. **(A)** At 12 hours post-induction KDM2B ChIPs were performed to test the binding of KDM2B on the RTA promoter. Cellular intergenic region (Neg) was used as a negative control. P-values are shown (n = 3). P<0.05 is considered to be statistically significant difference. **(B)** Immunoblot analysis of cell lysates collected at 0 and 12 hpi for the expression of KDM2B and viral proteins. Tubulin was used as a loading control. Asterisk indicates non-specific signal.
(TIF)

S7 Fig Testing the effect of KSHV infection on KDM2B expression. **(A)** Time course KSHV infection in SLK cells. The cells were mock infected or infected with KSHV BAC16 for 1, 2 or 3 days, and GFP images were taken to show the KSHV infected cells. **(B)** KDM2B gene expression was measured at the indicated post-infection time points by RT-qPCR.
(TIF)

S8 Fig KDM2B is not required for the recruitment of PRC1 to RTA promoter during *de novo* KSHV infection. **(A)** Immunoblots showing the expression of KDM2B and RING1B in shKDM2B-treated KSHV-infected SLK cells at 24 hpi. **(B)** ChIP assays testing the recruitment of PRC1 factor RING1B onto viral RTA promoter in the KDM2B depleted SLK cells infected with KSHV for 24 hours. **(C)** RING1B ChIP on Myc promoter. The cellular intergenic region Neg was used a negative control. (*p<0.05, statistically significant, ns: not significant).
(TIF)

S1 Table List of antibodies used in the study. (DOCX)

S2 Table Sequences of oligos used in the study. (DOCX)

S3 Table List of shRNA target sequences used for the inhibition of epigenetic factors. (DOCX)

S4 Table Summary of the siRNA screen results. (XLSX)
