# Supplementary figures and images for: Epigenetic factor siRNA screen during primary KSHV infection identifies novel host restriction factors for the lytic cycle of KSHV
Source: PLoS Pathog. 2020 Jan 10;16(1):e1008268. doi: 10.1371/journal.ppat.1008268 (PMC6977772; doi:10.1371/journal.ppat.1008268)

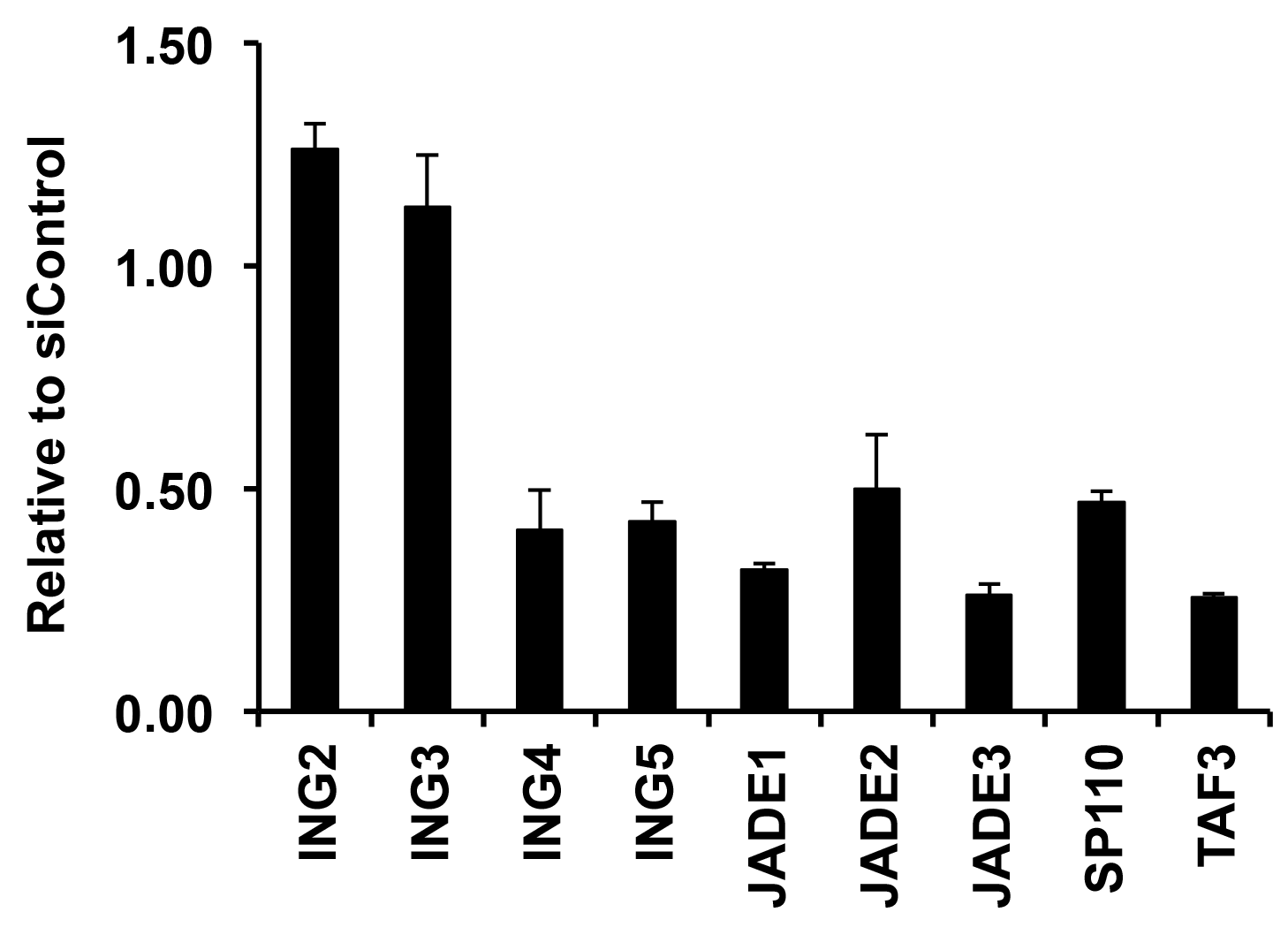

Supplement: S1 Fig — The viral DNA level was tested by qPCR at 60 hpi and calculated relative to the siControl sample. The host factors targeted by siRNAs are indicated along the x-axis. (TIF) [file ppat.1008268.s001.tif]

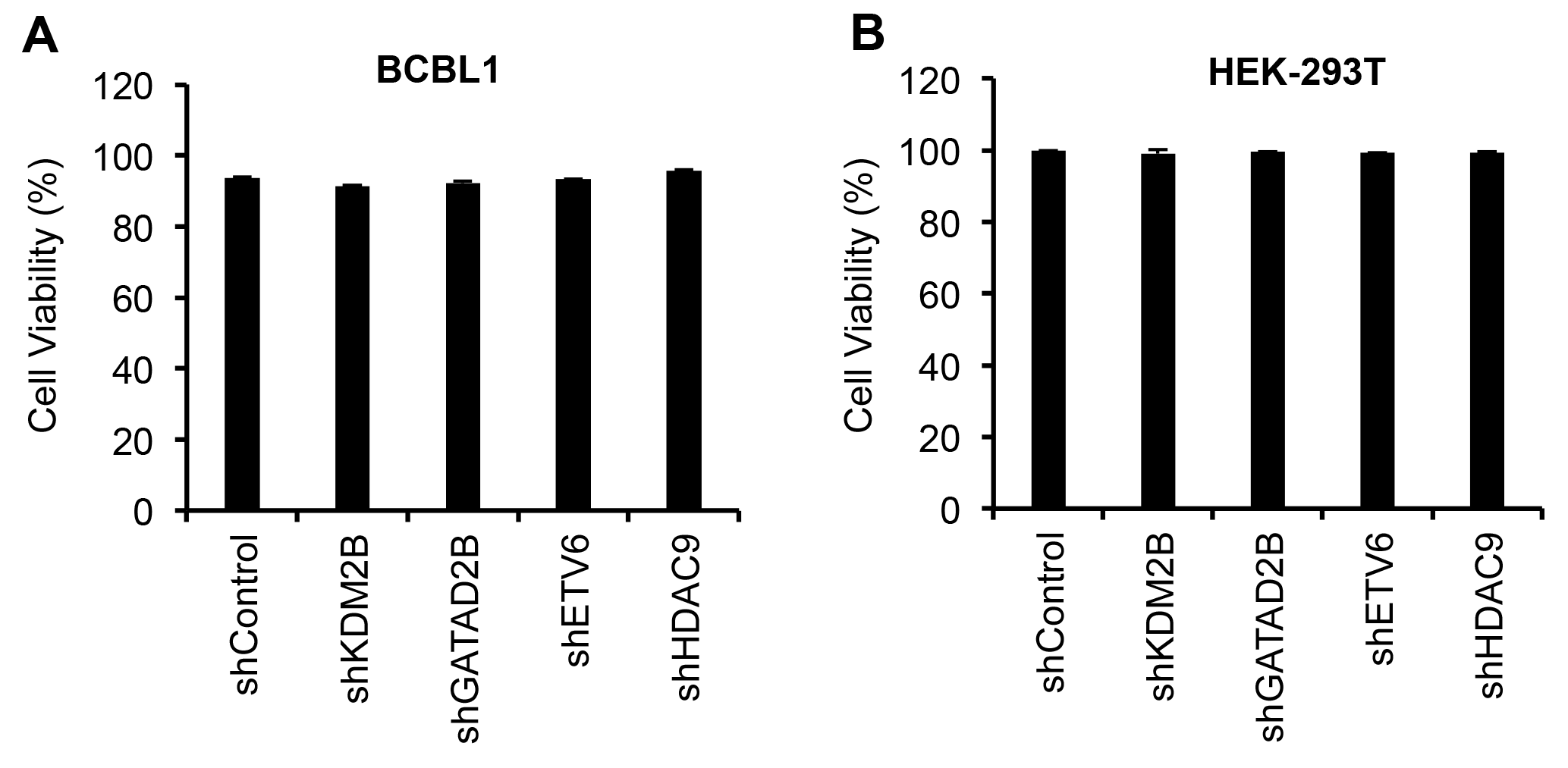

Supplement: S2 Fig — (A) Testing cell viability in latently infected BCBL1 cells following shRNA knockdown of host epigenetic factors. (B) Testing cell viability in HEK293T cells after shRNA knockdown of host epigenetic factors. (TIF) [file ppat.1008268.s002.tif]

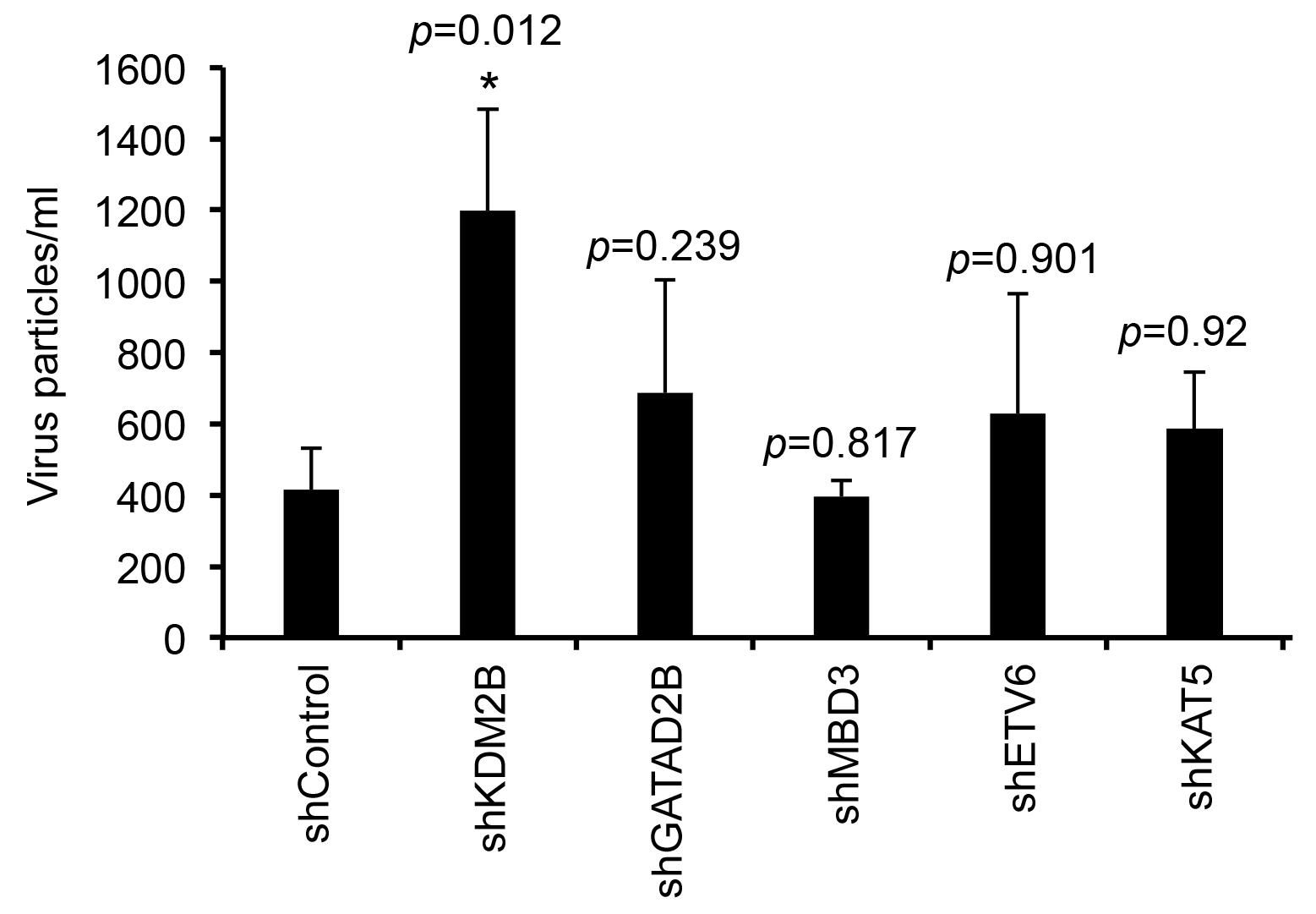

Supplement: S3 Fig — BCBL1 cells were infected with shRNA lentiviruses targeting the host epigenetic factors for 3 days and the viral DNA was isolated from the supernatant. The viral DNA was quantified by qPCR and the infectious virus particles were calculated. (TIF) [file ppat.1008268.s003.tif]

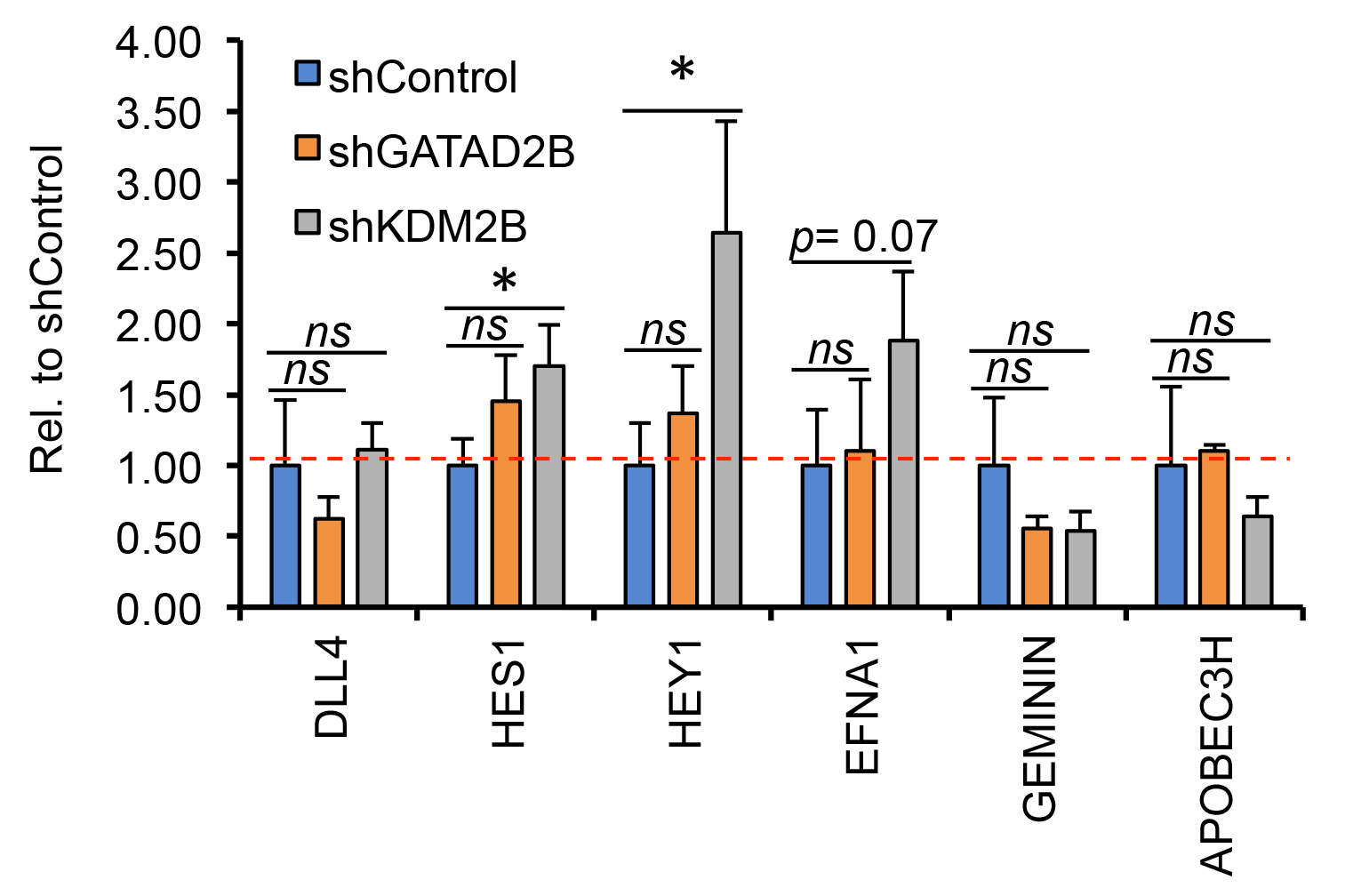

Supplement: S4 Fig — BCBL1 cells were infected with shRNA lentiviruses targeting GATAD2B or KDM2B for 3 days. The expression of host genes was analyzed by RT-qPCR and the fold change in gene expression was calculated relative to the shControl-treated sample (ns: not significant, asterisk indicates p<0.05). (TIF) [file ppat.1008268.s004.tif]

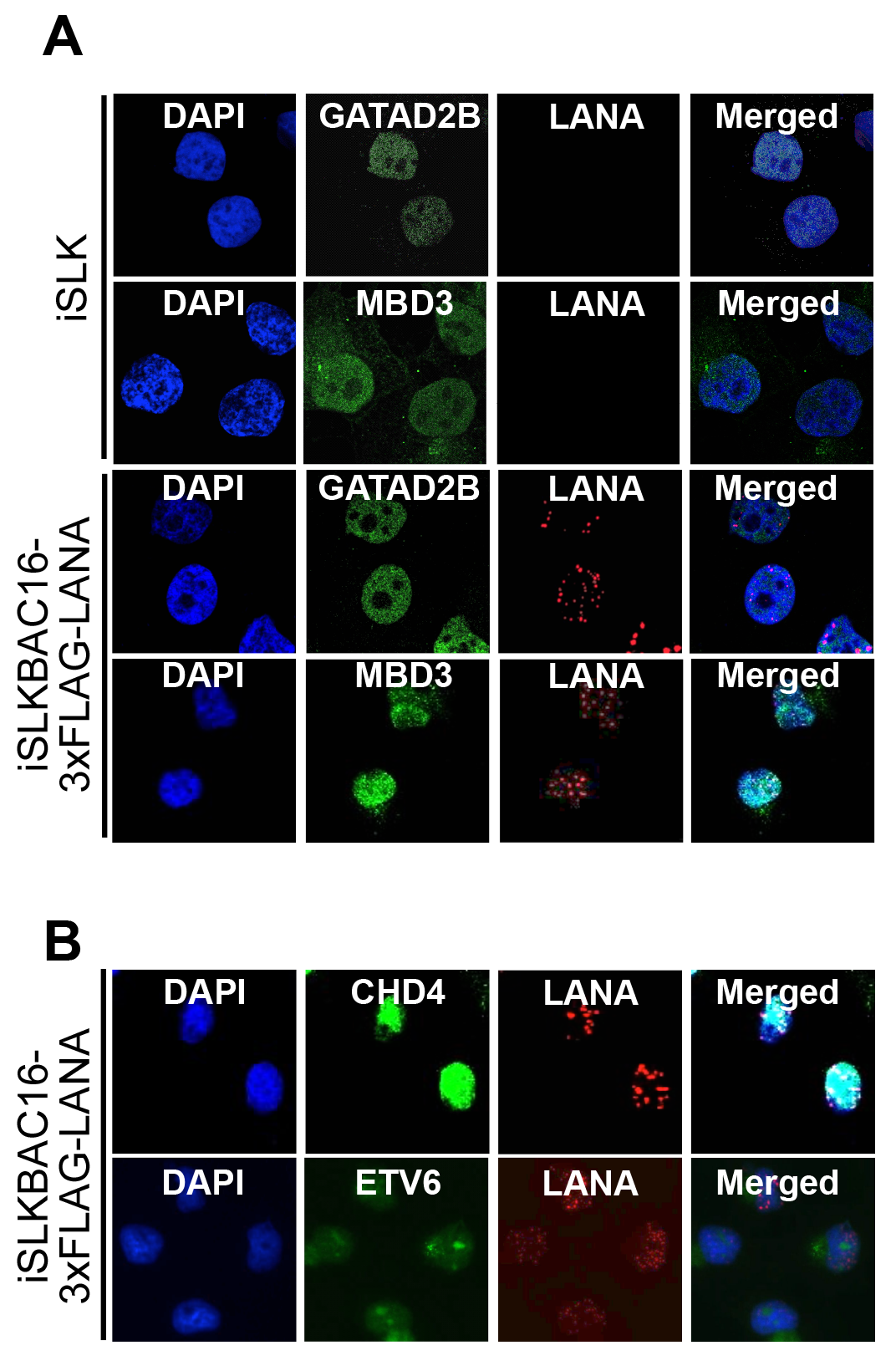

Supplement: S5 Fig — (A) Uninfected iSLK cells or KSHV-infected iSLK cells (iSLKBAC16-3xFLAG-LANA) were subjected to immunofluorescence analysis for LANA (red) and GATAD2B or MBD3 (green). (B) KSHV-infected iSLK cells (iSLKBAC16-3xFLAG-LANA) were subjected to immunofluorescence analysis for LANA (red) and CHD4 or ETV6 (green). FLAG antibody was used to detect 3xFLAG-LANA expressed from KSHV BAC16. (TIF) [file ppat.1008268.s005.tif]

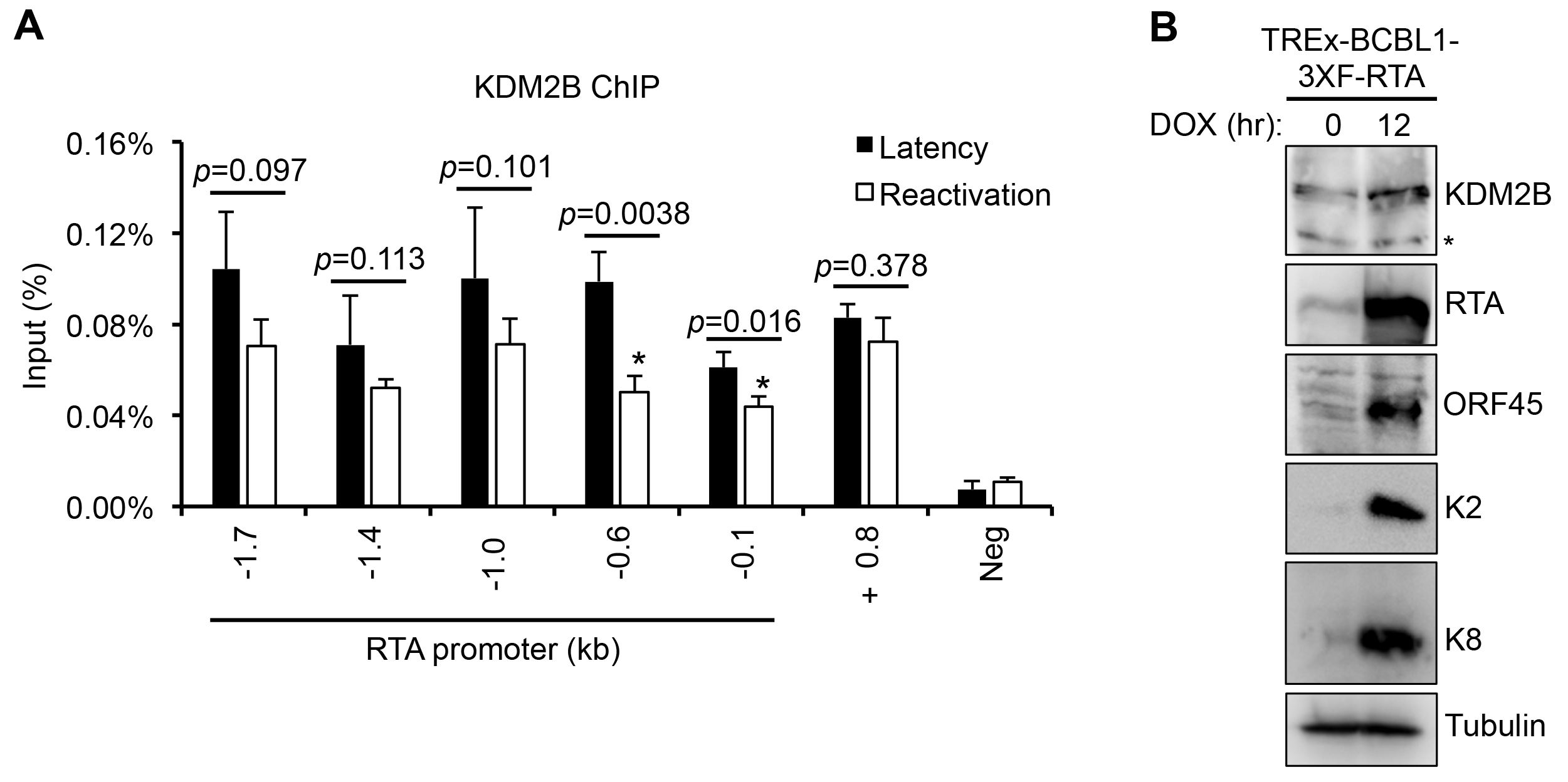

Supplement: S6 Fig — TRExBCBL1-3xFLAG-RTA cells were treated with 1 μg/ml doxycycline to induce the 3xFLAG-RTA transgene, which results in lytic reactivation. (A) At 12 hours post-induction KDM2B ChIPs were performed to test the binding of KDM2B on the RTA promoter. Cellular intergenic region (Neg) was used as a negative control. P-values are shown (n = 3). P<0.05 is considered to be statistically significant difference. (B) Immunoblot analysis of cell lysates collected at 0 and 12 hpi for the expression of KDM2B and viral proteins. Tubulin was used as a loading control. Asterisk indicates non-specific signal. (TIF) [file ppat.1008268.s006.tif]

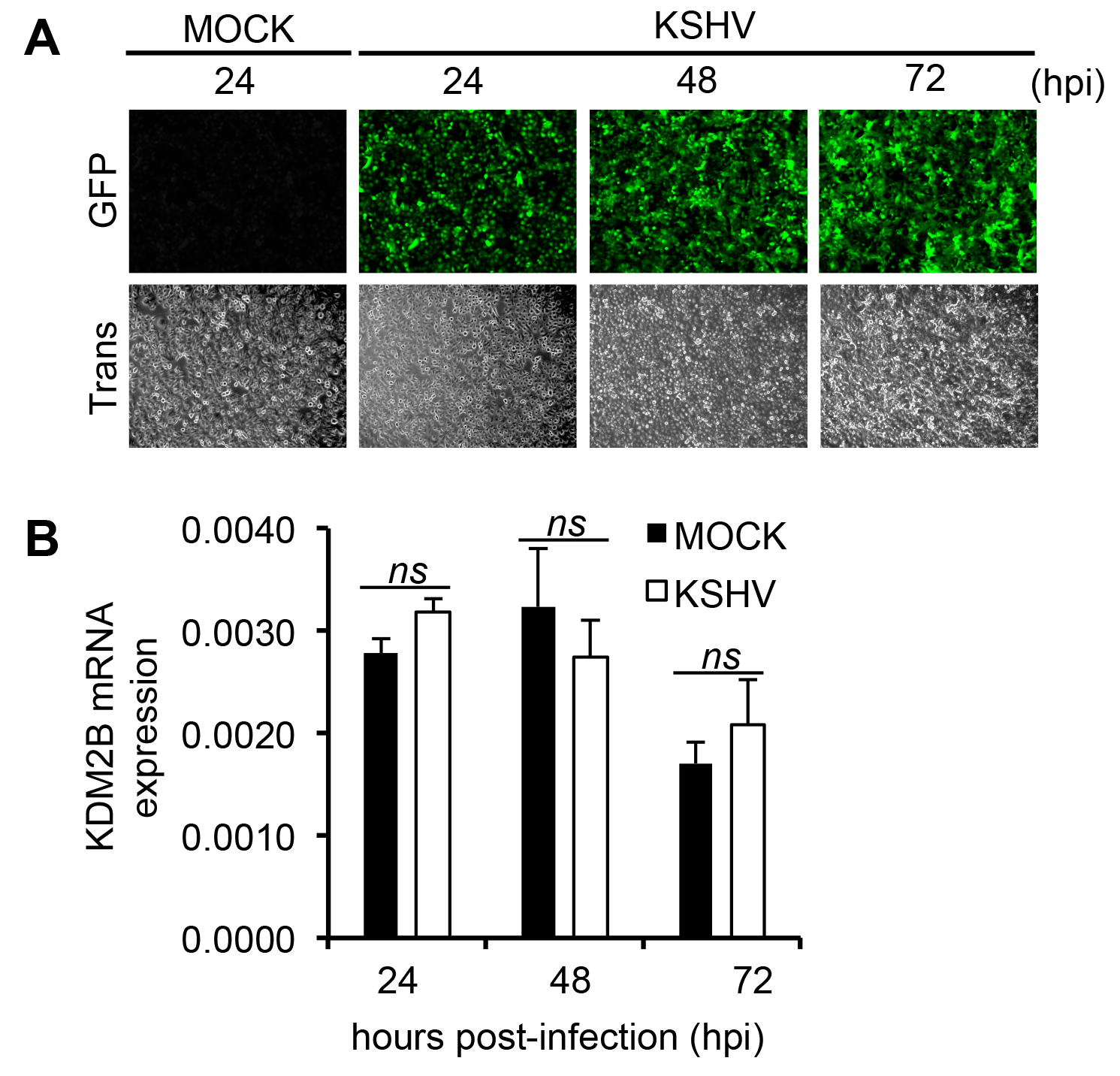

Supplement: S7 Fig — (A) Time course KSHV infection in SLK cells. The cells were mock infected or infected with KSHV BAC16 for 1, 2 or 3 days, and GFP images were taken to show the KSHV infected cells. (B) KDM2B gene expression was measured at the indicated post-infection time points by RT-qPCR. (TIF) [file ppat.1008268.s007.tif]

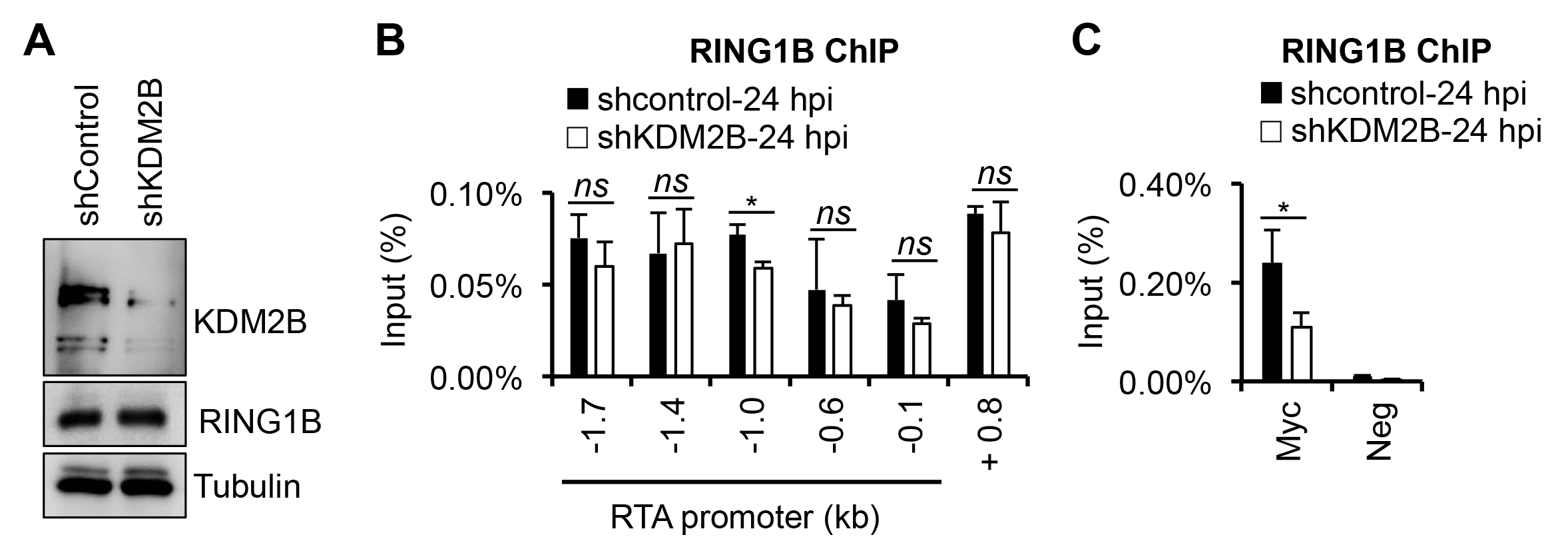

Supplement: S8 Fig — (A) Immunoblots showing the expression of KDM2B and RING1B in shKDM2B-treated KSHV-infected SLK cells at 24 hpi. (B) ChIP assays testing the recruitment of PRC1 factor RING1B onto viral RTA promoter in the KDM2B depleted SLK cells infected with KSHV for 24 hours. (C) RING1B ChIP on Myc promoter. The cellular intergenic region Neg was used a negative control. (*p<0.05, statistically significant, ns: not significant). (TIF) [file ppat.1008268.s008.tif]
