# Supplementary material for: Epigenetic factor siRNA screen during primary KSHV infection identifies novel host restriction factors for the lytic cycle of KSHV
Source: PLoS Pathog. 2020 Jan 10;16(1):e1008268. doi: 10.1371/journal.ppat.1008268 (PMC6977772; doi:10.1371/journal.ppat.1008268)
Supplement: S1 Table — (DOCX) [file ppat.1008268.s009.docx]

| Antibody | Host species | Dilution | Source |
| --- | --- | --- | --- |
| RTA | Rabbit polyclonal | 1/1000 (WB) | Yoshihiro Izumiya, UC Davis |
| ORF6 | Rabbit polyclonal | 1/1000 (WB); 1/400 (IF) | Gary S. Hayward,  Johns Hopkins University |
| ORF45 | Mouse monoclonal | 1/1000 (WB) | Santa Cruz; sc-53883 |
| K8 | Mouse monoclonal | 1/1000 (WB); 1/400 (IF) | Santa Cruz; sc-57889 |
| K8.1 | Mouse monoclonal | 1/1000 (WB) | Santa Cruz; sc-65446 |
| K2 | Rabbit polyclonal | 1/1000 (WB) | ABI; 13-214-050 |
| Tubulin | Mouse monoclonal | 1/5000 (WB) | Sigma; T5326 |
| MBD3 | Rabbit polyclonal | 1/1000 (WB); 1/50 (IF); 1-2 µg (ChIP) | Bethyl; A302-528A |
| GATAD2B | Rabbit polyclonal | 1/1000 (WB); 1/200 (IF); 1-2 µg (ChIP) | Bethyl; A301-283A |
| KDM2B | Rabbit polyclonal | 1/1000 (WB); 1/200 (IF); 1-2 µg (ChIP) | EMD Millipore; 09-864 |
| Normal IgG | Rabbit polyclonal | 1/50-1/400 (IF);  0.5-2 µg (ChIP) | Cell signaling; 2729S |
| Normal IgG | Mouse monoclonal | 1/500 (IF); 0.5-2 µg (ChIP) | Santa Cruz; sc-2025 |
| H3 | Rabbit polyclonal | 1 µg (ChIP) | Abcam; ab1791 |
| H3K27me3 | Rabbit polyclonal | 0.5 µg (ChIP) | Active Motif; 39155 |
| H3K4me3 | Rabbit polyclonal | 0.5 µg (ChIP) | Active Motif; 39159 |
| H3K36me2 | Rabbit polyclonal | 0.5 µg (ChIP) | Abcam; ab9049 |
| H3K79me2 | Rabbit polyclonal | 0.5 µg (ChIP) | Active Motif; 39143 |
| H3K4ac | Rabbit polyclonal | 0.5 µg (ChIP) | Active Motif; 39699 |
| H3K36ac | Rabbit polyclonal | 0.5 µg (ChIP) | Active Motif; 39281 |
| RYBP | Rabbit polyclonal | 2 µg (ChIP) | Abcam; ab5976 |
| RING1B | Rabbit polyclonal | 2 µg (ChIP) | Abcam; ab3832 |
| LANA | Rat polyclonal | 2 µg (ChIP) | Advanced Biotechnologies; 13-210-100 |
| FLAG | Mouse monoclonal | 1/1000 (WB); 1/500 (IF);  1 µg (ChIP) | Sigma; F1804 |

Table S1. List of antibodies used in this study.
