# Supplementary material for: Epigenetic factor siRNA screen during primary KSHV infection identifies novel host restriction factors for the lytic cycle of KSHV
Source: PLoS Pathog. 2020 Jan 10;16(1):e1008268. doi: 10.1371/journal.ppat.1008268 (PMC6977772; doi:10.1371/journal.ppat.1008268)
Supplement: S2 Table — (DOCX) [file ppat.1008268.s010.docx]

**Table S2: List of primers used in this study**

| **Gene** | **Forward (5' - 3')** | **Reverse (5' - 3')** | **Application** |
| --- | --- | --- | --- |
| **RTA (-1.7 kb)** | GATCGGCGAAGTGGATAGAGT | CCCTATTGGTCACATCTCACG | ChIP-qPCR |
| **RTA (-1.4 kb)** | TGAGGTCTATTTCCCACGACA | ACAGCTCCGACGATGAGTATG | ChIP-qPCR |
| **RTA (-1.0 kb)** | CCCCAACACAAGGACCTTTA | GCTTTTGGATACCCTGGTGA | ChIP-qPCR |
| **RTA (-0.6 kb)** | AAGACACTGACCCACCAAGG | GGTGCCACCAATGTATGACC | ChIP-qPCR |
| **RTA (-0.1 kb)** | AAAGTCAACCTTACTCCGCAAG | GCTGCCTGGACAGTATTCTCAC | ChIP-qPCR |
| **RTA (+0.8 kb)** | TTGCCAAGTTTGTACAACTGCT | ACCTTGCAAAGACCATTCAGAT | ChIP-qPCR, RT-qPCR |
| **Neg** | CAGGATCTCCGAGAATCAGC | GAGTTGGGAGAGCTGTCAGG | ChIP-qPCR |
| **K2pr** | CATACGCAGCCAAGCTATCA | GCTAGCACAGCAAATTGAGA | ChIP-qPCR |
| **K2gb** | TCACTGCGGGTTAATAGGATTT | CATGACGTCCACGTTTATCACT | ChIP-qPCR  RT-qPCR |
| **ORF25pr** | AGTTGTCGGTGTCTATCTGT | TGCAGAGCGATACGCAGACT | ChIP-qPCR |
| **ORF25gb** | ACAGTTTATGGCACGCATAGTG | GGTTCTCTGAATCTCGTCGTGT | ChIP-qPCR, RT-qPCR |
| **TR1** | GGGGGACCCCGGGCAGCGAG | GGCTCCCCCAAACAGGCTCA | ChIP-qPCR |
| **Myc** | TCCTTATGCCTCTATCATTCCTC | TGCAGGGCGCCTCGCTAAGGCT | ChIP-qPCR |
| **TR2** | CTCTCTCTACTGTGCGAGGAGT | TTCACGTAGTGTCCAGGGCTC | ChIP-qPCR |
| **ORF11** | GGCACCCATACAGCTTCTACGA | CGTTTACTACTGCACACTGCA | qPCR |
| **HS1** | TTCCTATTTGCCAAGGCAGT | CTCTTCAGCCATCCCAAGAC | qPCR |
| **ORF45** | CCATACAGCGACCCTGATGA | CCGATTCTCTGACTCAATACT | RT-qPCR |
| **ORF48** | CCACATCTTCATAGAGCACAT | ATTGCATCACCAGGGTATCCA | RT-qPCR |
| **K8** | TCTATGTAGTCGCCTCTTGGA | GGTCTGTGAAACGGTCATTGA | RT-qPCR |
| **ORF70** | ATGCAGGCCAGGTATAGTCT | TCCCATATCTTGACTCCTGT | RT-qPCR |
| **K5** | TAAGCACTTGGCTAACAGTGT | GGCCACAGGTTAAGGCGACT | RT-qPCR |
| **K6** | ATGCTGCGTTAGCGTACTGCT | GAACCCGTAGCAGCAGCTAT | RT-qPCR |
| **K7** | TGCCGCTTCACCTATGGATT | ACGCAATCAACCCACAATCG | RT-qPCR |
| **ORF36** | ATTGCCAACGACCTGATGCA | ACTCCAGTCCAGCTGCAGCA | RT-qPCR |
| **ORF44** | TGACCTAGAGTTCGATCCACTGGA | AAGATTCTGTGCCGCTACCACTGT | RT-qPCR |
| **ORF46** | GGTACGGGTCCTGGCCTAAGATCA | ACTGCTCTCGCTGCTCAGACAGAT | RT-qPCR |
| **ORF49** | GGCATGCTGCCACTAGTGGA | AGAGGCACCACTCTGTGCAG | RT-qPCR |
| **ORF56** | CACAGATTCCCGTCAATACAAA | GTATCTTCAGTAGGCGGCAGAG | RT-qPCR |
| **ORF57** | AGGGATATCACCGCTCTCATAAGA | CTGCGGTTTCTCGACGGCAACTCA | RT-qPCR |
| **vIRF1** | TAAACGAGGCGTCCACGGGCATGT | CCGGAGTAGAGTGGGAAGACGAAG | RT-qPCR |
| **vIRF2** | TCATGGCTGGTTCCTGCGTCAAGT | AGGACCGCCAATCGAGCCAGACAG | RT-qPCR |
| **vIRF3** | CGAGCCGTACACTGTGTTGATACA | CTTAGACGCTTGCCAGGTGAAGAG | RT-qPCR |
| **vIRF4** | AGTGTCACTGCGTCGCGTAGCCGT | ACATTTGGTGGTAGCTACGTAGTA | RT-qPCR |
| **ORF59** | AACCGCAGTTCGTCAGGACCACCA | CCTTAGCCACTTAAGTAGGAATG | RT-qPCR |
| **ORF18** | TGTGGAAGCTCGTGTACGAT | TATCGTTCAAGTGCATCCAG | RT-qPCR |
| **ORF23** | GCACGCTCCATGATGGTAAC | CGTGTCACTTAACACAGACA | RT-qPCR |
| **ORF33** | ATCATGTACGTCGTAAGCCA | GAAATCTCAGTCCGGATGCT | RT-qPCR |
| **ORF39** | AGTAGTATACTATGGCAATGCCGT | GTAAACTACAGCGCGCTAAACCTCA | RT-qPCR |
| **ORF42** | CTGGTGGAGCACGAAGACAT | CGATAACAGGCAGCAGAGCT | RT-qPCR |
| **ORF64** | CTTCCTCGAGGGCATCATATAC | TATACGGTGATGGACTTGATGG | RT-qPCR |
| **ORF68** | CCAACTCTCAACTCACGATC | CGACTGGTGAATCTGTGTCA | RT-qPCR |
| **18S** | TTCGAACGTCTGCCCTATCAA | GATGTGGTAGCCGTTTCTCAGG | RT-qPCR |
| **LANA** | GAGTCTGGTGACGACTTGGAG | AGGAAGGCCAGACTCTTCAAC | RT-qPCR |
| **RTA** | CAAGGTGTGCCGTGTAGAGAT | GGTCAAAGCCTTACGCTTCTT | RT-qPCR |
| **GATAD2B** | ATCAATGATGAGCCTGTGGATA | AATGATGTCTGGTGAGGGAGTT | RT-qPCR |
| **KAT5** | AAATCGAATTGTTTGGGCACT | TGGCTTCGATCAGACACCAG | RT-qPCR |
| **KDM2B** | AGTCCGAGACGTCAAACTCCTA | TAGTAACGCACAAACTGGGACA | RT-qPCR |
| **ETV6** | GCTCAGGATGAGGAAGACTC | GAAAACTCATTTTCAGCCCACT | RT-qPCR |
| **MBD5** | TGTGGGAAGTAGACATTGAAGG | GGGTTCTATGGATTGATGAAGG | RT-qPCR |
| **HDAC9** | AAGGAGCAGAAACTGGAGCAG | CGTCCTCTATCTTTGCCTCTG | RT-qPCR |
