# Supplementary material for: Epigenetic factor siRNA screen during primary KSHV infection identifies novel host restriction factors for the lytic cycle of KSHV
Source: PLoS Pathog. 2020 Jan 10;16(1):e1008268. doi: 10.1371/journal.ppat.1008268 (PMC6977772; doi:10.1371/journal.ppat.1008268)
Supplement: S3 Table — (DOCX) [file ppat.1008268.s011.docx]

**Table S3. List of shRNA target sequence used for lentivirus production.**

| **Target gene** | **shRNA target sequence 5’ to 3’** |
| --- | --- |
| **KDM2B** | GCATGAAGCAGAGCTGCATCA |
| **GATAD2B** | GGATAGAATGACAGAAGATGC |
| **MBD3** | GCAAGATGCTGATGAGCAAGA |
| **KAT5** | CGTCCATTACATTGACTTCAA |
| **ETV6** | GTTTCCTTCTGATTTGGAA |
| **HDAC9** | CCATCCTACAAGTACACATTA |
